# MiR‐181a‐5p promotes neural stem cell proliferation and enhances the learning and memory of aged mice

**DOI:** 10.1111/acel.13794

**Published:** 2023-02-16

**Authors:** Qiaoyi Sun, Li Ma, Jing Qiao, Xing Wang, Jianguo Li, Yuxi Wang, Ailing Tan, Zihui Ye, Yukang Wu, Jiajie Xi, Jiuhong Kang

**Affiliations:** ^1^ Clinical and Translational Research Center of Shanghai First Maternity and Infant Hospital, Shanghai Key Laboratory of Maternal Fetal Medicine, Shanghai Key Laboratory of Signaling and Disease Research, Frontier Science Center for Stem Cell Research, National Stem Cell Translational Resource Center, School of Life Sciences and Technology Tongji University Shanghai China

**Keywords:** aging, hippocampus, miR‐181a‐5p, neural stem cells, PTEN

## Abstract

Hippocampal neural stem cell (NSC) proliferation is known to decline with age, which is closely linked to learning and memory impairments. In the current study, we found that the expression level of miR‐181a‐5p was decreased in the hippocampal NSCs of aged mice and that exogenous overexpression of miR‐181a‐5p promoted NSC proliferation without affecting NSC differentiation into neurons and astrocytes. The mechanistic study revealed that phosphatase and tensin homolog (PTEN), a negative regulator of the AKT signaling pathway, was the target of miR‐181a‐5p and knockdown of PTEN could rescue the impairment of NSC proliferation caused by low miR‐181a‐5p levels. Moreover, overexpression of miR‐181a‐5p in the dentate gyrus enhanced the proliferation of NSCs and ameliorated learning and memory impairments in aged mice. Taken together, our findings indicated that miR‐181a‐5p played a functional role in NSC proliferation and aging‐related, hippocampus‐dependent learning and memory impairments.

## Graphical abstract

Sun et al. report that miR‐181a‐5p is downregulated in the hippocampus NSCs of aged mice and targets PTEN to regulate NSC proliferation. Overexpression of miR‐181a‐5p in the dentate gyrus enhances NSC proliferation and ameliorates learning and memory impairments in aged mice.

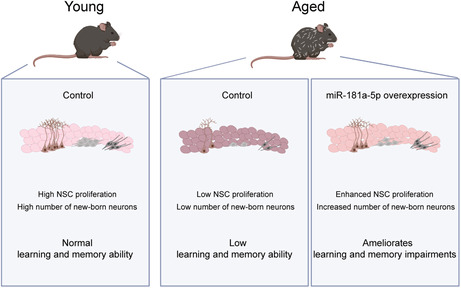

## 1 INTRODUCTION

Aging is a progressive degenerative state that is often accompanied by cognitive deterioration and memory deficits. The hippocampus is a brain region closely related to spatial learning and memory. Adult neural stem cells (NSCs), which are located in the subgranular zone (SGZ) of the dentate gyrus (DG) in the hippocampus, play important roles in the function of the hippocampus (Ma et al., 2009). NSCs have the ability to proliferate, either generating two NSCs to maintain NSC pools through symmetric division or generating one NSC and one neural progenitor cell (NPC) through asymmetric division. NPCs then further differentiate into particular cell types, such as neurons or astrocytes (Bonaguidi et al., 2011; Bond et al., 2015; Temple, 1989). Studies on rodents have shown that proliferation and neurogenesis of NSCs persist throughout the lifespan; however, adult neurogenesis decreases with age (Ben Abdallah et al., 2010; Kuhn et al., 1996), and this decrease is involved in cognitive and memory declines, indicating that abnormalities in hippocampal NSCs are one of the main causes of age‐related deterioration in hippocampus‐dependent cognition (Berdugo‐Vega et al., 2020; Drapeau & Abrous, 2008). Whether adult hippocampal neurogenesis persists in adult and aged humans and whether there is sufficient generation of neurons in adult humans to contribute to brain function remains extensively debated due to the time interval of postmortem sampling and the difference in tissue processing methods (Kempermann et al., 2018; Moreno‐Jimenez et al., 2019; Sorrells et al., 2018). Therefore, understanding the molecular mechanisms underlying aging‐related hippocampal NSC abnormalities is important for developing therapeutic strategies to overcome aging‐related cognitive deterioration and memory deficits.

Proliferation and neurogenesis of NSCs are exquisitely regulated by extrinsic and intrinsic factors (Bond et al., 2015; Navarro Negredo et al., 2020), including secreted molecules, neurotransmitters, transcription factors (Ahmad et al., 2019), and epigenetic regulators (Wang et al., 2018). Among these, miRNAs, which are enriched in the brain, have been shown to be widely involved in the regulation of NSC proliferation and differentiation (Lopez‐Ramirez & Nicoli, 2014; Walgrave et al., 2021). MiR‐218‐2 targets complement component 3 (C3) to regulate presynaptic glutamate release and synaptic plasticity in mouse hippocampal neurons and then bidirectionally regulates learning and memory in mice (Lu, Fu, et al., 2021). Increased expression of miRNA let‐7b during aging could repress Hmga2 expression and contribute to declining self‐renewal of NSCs (Tzatsos & Bardeesy, 2008). Our previous study also found that MiR‐153 promoted neurogenesis and increased the cognitive ability of aged mice by directly targeting the Notch signaling pathway key factors Jag1 and Hey2 (Qiao et al., 2020). Together, these studies clearly indicate that miRNAs play important roles in NSC proliferation and differentiation, whereas the function of many miRNAs that are highly expressed in NSCs is still unclear.

Phosphatase and tensin homolog (PTEN) is a lipid and protein phosphatase that acts as a negative regulator of the phosphatidylinositol‐3 kinase (PI3K)‐AKT pathway. Previous studies have shown that PTEN plays critical roles in regulating tumor cell proliferation, migration, and death (Cristofano et al., 1998; He et al., 2007). PTEN also regulates the neural system. Mice lacking PTEN exhibited increased cell proliferation, decreased cell death, and enlarged cell sizes, resulting in enlarged, histoarchitecturally abnormal brains (Groszer et al., 2001). Loss of PTEN enhances the self‐renewal capacity of NSCs while maintaining multilineage cell differentiation potential (Groszer et al., 2001, 2006). Although PTEN plays important roles in NSC self‐renewal and proliferation, whether it influences NSC proliferation and learning and memory deficits in the aged hippocampus is still unclear.

In this study, we found that miR‐181a‐5p is downregulated in the hippocampal NSCs of aging mice and mediates the aging‐related, hippocampus‐dependent impairments of learning and memory. Moreover, overexpression of miR‐181a‐5p in the aged mouse hippocampus could ameliorate its impaired cognitive abilities.

## 2 RESULTS

### 2.1 MiR‐181a‐5p decreases with aging in the mouse hippocampus

**FIGURE 1:**
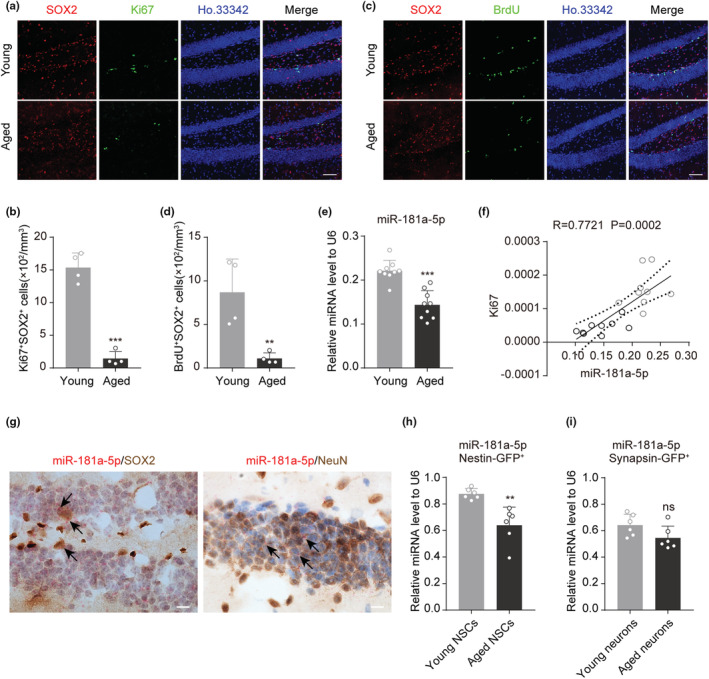
MiR‐181a‐5p decreases in the hippocampi of aged mice. (a,b) Representative images of Ki67 (green), SOX2 (red), and Ho.33342 (blue) in the DG of young and aged mice (a) and quantification of the number of Ki67^+^SOX2^+^Ho.33342^+^ cells in the DG (b). Scale bars, 50 μm (*n* = 4 per group). (c,d) Representative images of BrdU (green), SOX2 (red), and Ho.33342 (blue) in the DG of young and aged mice (c) and quantification of the number of BrdU^+^SOX2^+^Ho.33342^+^ cells in the DG (d). Scale bars, 50 μm (*n* = 4 per group). (e) qRT–PCR analysis of the expression of miR‐181a‐5p in the hippocampus of young and aged mice. U6 was used as the internal control (*n* = 9 per group). (f) The correlation analysis for miR‐181a‐5p and Ki67. (g) Representative images of miR‐181a‐5p in situ hybridization coupled to SOX2 or NeuN immunohistochemical staining in the dentate gyrus of 3‐month‐old WT mice. Black arrows show miR‐181a‐5p positive dots. Scale bars: 5 μm. (h) qRT–PCR analysis of the expression of miR‐181a‐5p in Nestin‐GFP^+^ populations sorted from the dentate gyrus of young and aged mice (*n* = 6 per group). (i) qRT–PCR analysis of the expression of miR‐181a‐5p in Syn‐GFP^+^ populations sorted from the dentate gyrus of young and aged mice (*n* = 6 per group). *p < 0.05, **p < 0.01, ***p < 0.001, ns: not significant. Values are presented as mean ± SD. Student's t test was used in (b), (d), (e), (h), and (i). Pearson correlation test was used in (f). See also Figure S1.

To evaluate the learning and memory abilities of aged mice, we conducted behavioral tests: the novel object recognition (NOR) test and the Morris water maze (MWM) test (Figure S1a). In the NOR test, no obvious differences in recognition of the two objects were observed between young and aged mice (Figure S1b), but the discrimination index and the discrimination ratio of novel objects were reduced in the aged mice (Figure S1c,d). In the MWM test, compared with young mice, aged mice took longer to locate the hidden platform, crossed the hidden platform fewer times, and spent less time in the goal quadrant (Figure S1e–h), although there was no significant difference in swimming speed or distance between the two groups (Figure S1i,j). When we detected the proliferation abilities of NSCs in the mouse hippocampus, we found that the expression of Ki67 and the number of Ki67^+^/Sox2^+^ NSCs decreased in aged mice (Figure S1k, Figure 1a,b). In BrdU incorporation analysis, the number of BrdU^+^/Sox2^+^ cells, which indicates the number of proliferating NSCs during the period of BrdU administration (Figure S1a), also decreased in aged mice (Figure 1c,d). These results demonstrate that the proliferation ability of hippocampal NSCs decreased with aging.

As miRNAs play key roles in the regulation of NSC proliferation, we analyzed differentially expressed miRNAs in the whole brains of young and aged mice (GSE34393) with the top 20 highly expressed miRNAs in the hippocampus of mice (GSE107496) and identified 4 miRNAs, let‐7f‐5p, miR‐101a‐3p, miR‐181a‐5p, and miR‐127‐3p, as the candidates (Figure S1l). Among these 4 miRNAs, only miR‐181a‐5p was decreased in the hippocampus of aged mice (Figure 1e and S1m), and its expression level was positively correlated with the extent of Ki67 expression (Figure 1f). These data indicate that miR‐181a‐5p deficiency may be associated with the decreased proliferation ability of aged hippocampal NSCs.

We next assessed miR‐181a‐5p localization in the dentate gyrus. We employed in situ hybridization in conjunction with cell‐specific markers to label NSCs, neurons, and astrocytes, hybridization with RNU6 and scramble probes were used as positive and negative controls, respectively (Figure S1n). The results showed that miR‐181a‐5p is mainly expressed in SOX2^+^ NSCs and NEUN^+^ neurons (Figure 1g). To evaluate the age‐related changes of miR‐181a‐5p in NSCs and neurons, we injected rAAVs containing Nestin promoter‐driven green fluorescent protein (Nestin‐GFP) or Synapsin promoter‐driven green fluorescent protein (Syn‐GFP) into the hippocampus and used FACS to isolate GFP‐positive cells from the dentate gyrus of young and aged mice. The qRT–PCR assay showed that miR‐181a‐5p was decreased in the Nestin‐GFP^+^ NSCs of aged mice (Figure 1h) but unchanged in Syn‐GFP^+^ neurons (Figure 1i), indicating that miR‐181a‐5p may mainly play its regulatory role in NSCs.

### 2.2 MiR‐181a‐5p promotes NSC proliferation without affecting its differentiation potential

**FIGURE 2:**
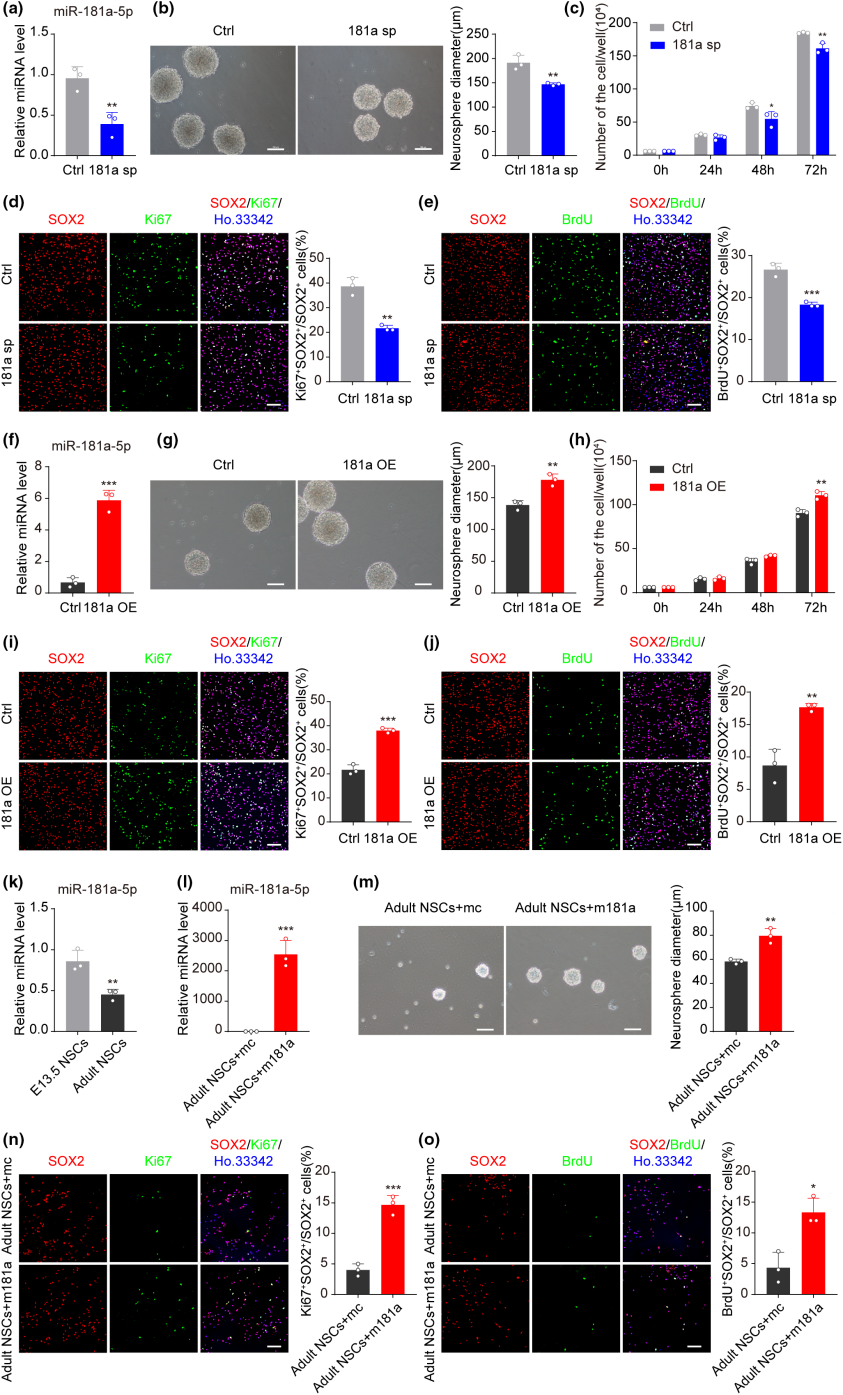
MiR‐181a‐5p is important for NSC proliferation. (a) qRT–PCR analysis the expression of miR‐181a‐5p in early‐passage NSCs infected with control (ctrl) or miR‐181a‐5p sponge (181 sp) viruses. U6 was used as the internal control. (b) Representative images of early‐passage neurospheres infected with ctrl or 181a sp viruses (left) and quantification of neurosphere diameters (right). Scale bars, 100 μm. (c) Number of cells at 24 h, 48 h, and 72 h after early‐passage NSCs were infected with ctrl or 181a sp viruses. (d) Representative images of Ki67 (green) and SOX2 (red) after early‐passage NSCs were infected with ctrl or 181a sp viruses (left) and the proportion of Ki67^+^SOX2^+^ cells among all SOX2^+^ cells (right). Scale bars, 100 μm. (e) Representative images of BrdU (green) and SOX2 (red) after early‐passage NSCs were infected with ctrl or 181a sp viruses (left) and the proportion of BrdU^+^SOX2^+^ cells among all SOX2^+^ cells (right). Scale bars, 100 μm. (f) qRT–PCR analysis of the overexpression efficiency of miR‐181a‐5p after late‐passage NSCs were infected with control (ctrl) or miR‐181a‐5p overexpression (181a OE) viruses. U6 was used as the internal control. (g) Representative images of late‐passage neurospheres infected with ctrl or 181a OE viruses (left) and quantification of neurosphere diameters (right). Scale bars, 100 μm. (h) Number of cells at 24 h, 48 h, and 72 h after late‐passage NSCs were infected with ctrl or 181a OE viruses. (i) Representative images of Ki67 (green) and SOX2 (red) after late‐passage NSCs were infected with ctrl or 181a OE viruses (left) and the proportion of Ki67^+^SOX2^+^ cells among all SOX2^+^ cells (right). Scale bars, 100 μm. (j) Representative images of BrdU (green) and SOX2 (red) after late‐passage NSCs were infected with ctrl or 181a OE viruses (left) and the proportion of BrdU^+^SOX2^+^ cells among all SOX2^+^ cells (right). Scale bars, 100 μm. (k) qRT–PCR analysis the expression of miR‐181a‐5p in E13.5 NSCs and adult hippocampal NSCs. U6 was used as the internal control. (l) qRT–PCR analysis the expression of miR‐181a‐5p after adult hippocampal NSCs were transfected with control mimics (mc) or miR‐181a‐5p mimics (m181a). U6 was used as the internal control. (m) Representative images of adult hippocampal neurospheres transfected with mc or m181a (left) and quantification of neurosphere diameters (right). Scale bars, 100 μm. (n) Representative images of Ki67 (green) and SOX2 (red) after adult hippocampal NSCs were transfected with mc or m181a (left), and the proportion of Ki67^+^SOX2^+^ cells among all SOX2^+^ cells (right). Scale bars, 100 μm. (o) Representative images of BrdU (green) and SOX2 (red) after adult hippocampal NSCs were transfected with mc or m181a (left), and the proportion of BrdU^+^SOX2^+^ cells among all SOX2^+^ cells (right). Scale bars, 100 μm. (a–e) were tested in early‐passage NSCs; (f–j) were tested in late‐passage NSCs; (l–o) were tested in adult hippocampal NSCs. Nuclei were stained with Hoechst 33342 (blue), and “merge” images indicate the merging of images acquired with distinct channels (green, red, and blue). *p < 0.05, **p < 0.01, ***p < 0.001. Values are presented as mean ± SD. Student's t test was used in (a), (b), (d–g), and (i–o), while two‐way ANOVA with Tukey's post hoc test for multiple comparisons was applied in (c) and (h). See also Figures S2 and S3.

To determine the role of miR‐181a‐5p in NSCs, we isolated NSCs from the forebrains of E13.5 mice for in vitro experiments. Following culture, the percentage of Ki67/Sox2 double‐positive cells decreased in late‐passage NSCs compared with early‐passage NSCs (Figure S2a). The BrdU incorporation analysis showed similar results (Figure S2b). These experiments indicate that the proliferation ability of NSCs decreased following passage. In addition, the expression of miR‐181a‐5p in NSCs was also decreased following passage (Figure S2c). We then constructed a ubiquitin promoter‐miR‐181a‐5p sponge‐WPRE lentivirus (181a sp) and infected early‐passage NSCs. The infect effect was validated by flow cytometry (Figure S2d). The expression level of miR‐181a‐5p was validated by qRT–PCR (Figure 2a). The expression of the miR‐181a‐5p sponge in early‐passage NSCs resulted in smaller neurospheres and a low cell proliferation rate (Figure 2b,c). The percentage of Ki67/Sox2 or BrdU/Sox2 double‐positive cells was also decreased in the 181a sp group (Figure 2d,e). To further confirm the inhibitory effect of miR‐181a‐5p on NSC proliferation, we transfected early‐passage NSCs with a miR‐181a‐5p inhibitor. The transfected effect was validated by flow cytometry (Figure S2e), and the inhibitory efficiency of miR‐181a‐5p inhibitor was validated by qRT–PCR (Figure S2f). Similar results to those of the 181a sp group in the neurosphere diameters and the cell proliferation curve, as well as the percentages of BrdU/Sox2 and Ki67/Sox2 double‐positive cells were observed in miR‐181a‐5p inhibitor group (Figure S2g–j).

Then, we constructed an overexpression lentivirus (181a OE) and infected late‐passage NSCs. The expression level of miR‐181a‐5p increased in the 181a OE group (Figure 2f), and this expression resulted in larger neurospheres and a higher proliferation rate in late‐passage NSCs (Figure 2g,h). The percentage of Ki67/Sox2 or BrdU/Sox2 double‐positive cells was also increased in the 181a OE group (Figure 2i,j). Similar results were obtained when late‐passage NSCs were transfected with miR‐181a‐5p mimics (Figure S2k–o). In addition, annexin/PI staining analysis showed that neither overexpression nor inhibition of miR‐181a‐5p induced NSC apoptosis (Figure S3a–d).

To further confirm the effect of miR‐181a‐5p on NSC proliferation, we isolated adult hippocampal NSCs. The expression level of miR‐181a‐5p was lower in the adult hippocampal NSCs than in the E13.5 NSCs (Figure 2k). Similar as in E13.5 NSCs, transfecting adult hippocampal NSCs with miR‐181a‐5p mimics resulted in larger neurospheres and a higher percentage of Ki67/Sox2 or BrdU/Sox2 double‐positive cells (Figure 2l–o).

**FIGURE 3:**
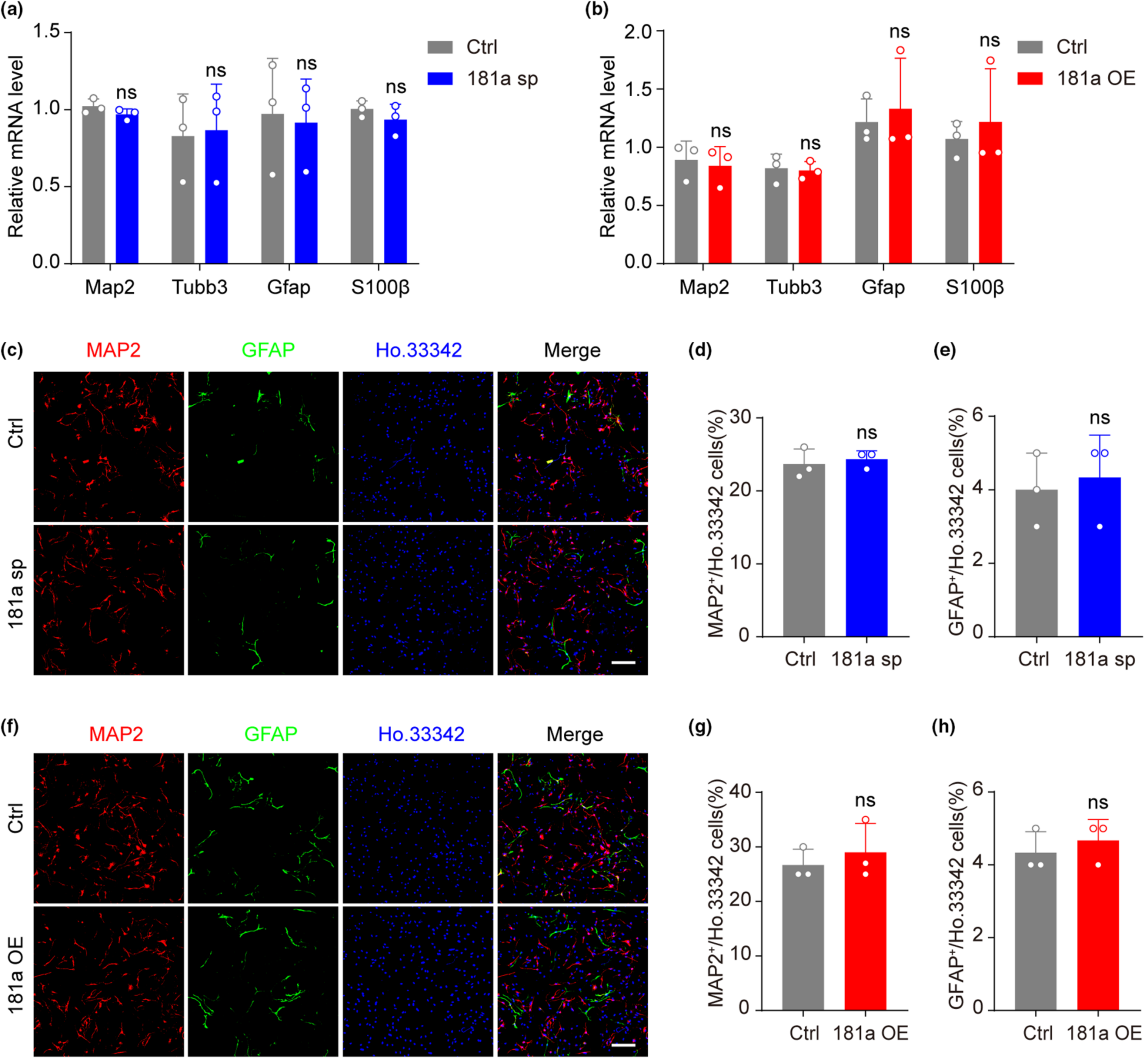
MiR‐181a‐5p does not affect NSC differentiation potential. (a) qRT–PCR analysis of the expression of neuron‐related genes (Map2 and Tubb3) and astrocyte‐related genes (Gfap and S100β) in NSCs infected with control (ctrl) or miR‐181a‐5p sponge (181a sp) viruses after differentiation. GAPDH was used as the internal control. (b) qRT–PCR analysis of the expression of neuron‐related genes (Map2 and Tubb3) and astrocyte‐related genes (Gfap and S100β) in NSCs infected with control (ctrl) or miR‐181a‐5p overexpression (181a OE) viruses after differentiation. GAPDH was used as the internal control. (c–e) Representative images of GFAP (green) and MAP2 (red) in NSCs infected with ctrl or 181a sp viruses after differentiation (c) and quantification of MAP2^+^ cells (d) and GFAP^+^ cells (e). Scale bars, 100 μm. (f–h) Representative images of GFAP (green) and MAP2 (red) in NSCs infected with ctrl or 181a OE viruses after differentiation (f) and quantification of MAP2^+^ cells (g) and GFAP^+^ cells (h). Scale bars, 100 μm. The 9th to 12th passages were used for experiments. ns: not significant. Values are presented as mean ± SD. Two‐way ANOVA with Tukey's post hoc test for multiple comparisons was used in (a) and (b), while Student's t test was used in (d), (e), (g), and (h).

Previous studies have shown that a few miRNAs, such as miR‐195 and miR‐184, can promote NSC proliferation but inhibit its differentiation potential (Liu et al., 2010, 2013). To determine whether miR‐181a‐5p affects the differentiation of NSCs, the neurospheres were dissociated into single cells and attached to glass coverslips for 48 h. qRT–PCR showed that neither overexpression of the miR‐181a‐5p sponge nor overexpression of miR‐181a‐5p affected the mRNA levels of the neuron‐related genes Map2 and Tubb3 or the astrocyte‐related genes GFAP and S100β (Figure 3a,b). Immunofluorescence staining also showed that it had no difference in the percentage of MAP2^+^ or GFAP^+^ cells in the control, 181a sp group (Figure 3c–e) and 181a OE group (Figure 3f–h). These data indicate that miR‐181a‐5p can promote NSC proliferation and does not affect its differentiation potential.

### 2.3 PTEN is the functional target of miR‐181a‐5p in NSC proliferation

**FIGURE 4:**
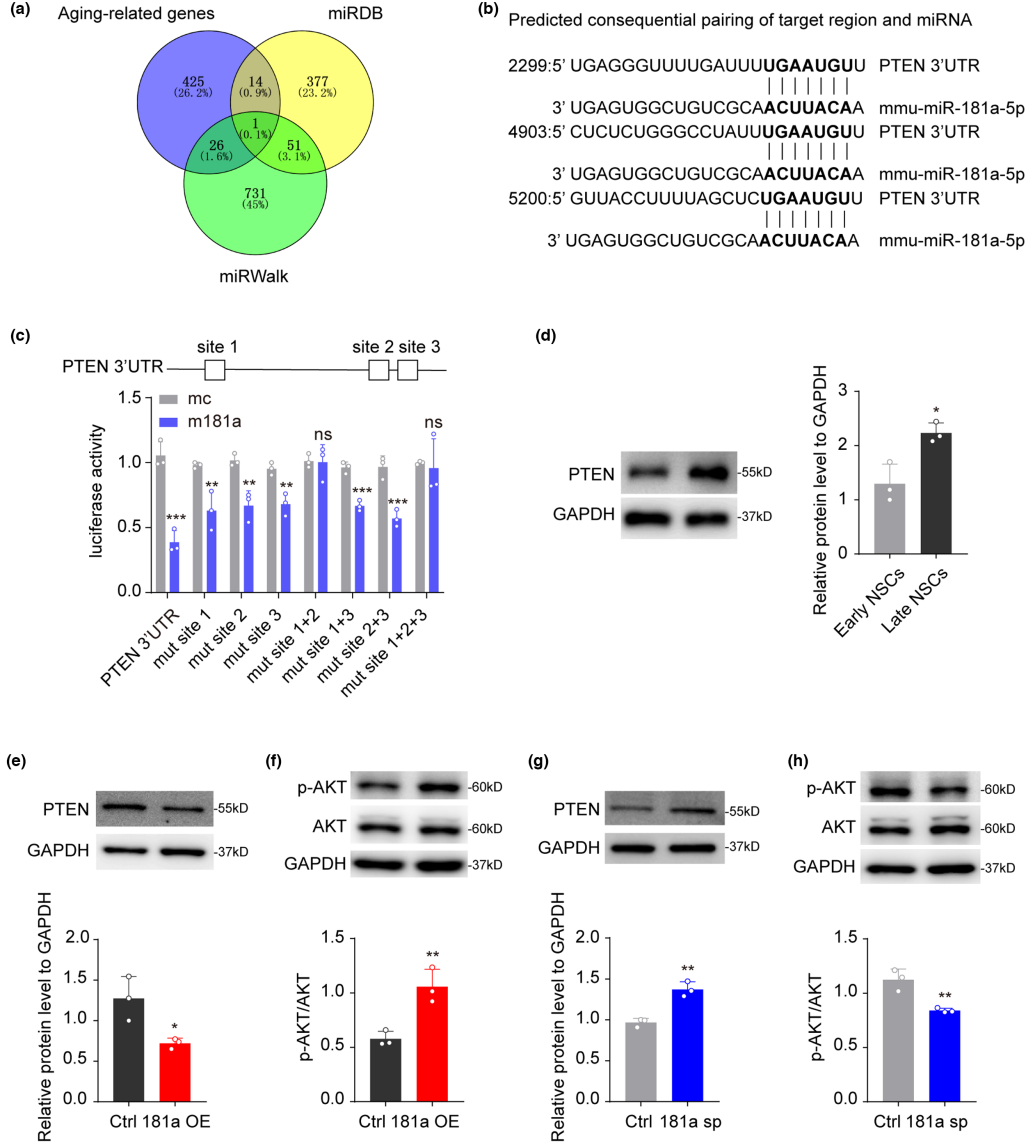
PTEN is a target of miR‐181a‐5p. (a) Venn diagrams showing the overlap of genes associated with aging and candidate miR‐181a‐5p target genes from miRDB and miRWalk. (b) The binding sequences of miR‐181a‐5p and the 3'UTR of Pten. (c) Luciferase reporter assays for miR‐181a‐5p and the full‐length Pten 3'UTR with native or mutant binding sites. (d) Western blot analysis of PTEN expression in early‐passage NSCs (early NSCs) and late‐passage NSCs (late NSCs). (e,f) Western blot analysis of PTEN (e), phospho‐AKT (p‐AKT) and AKT (f) expression in late‐passage NSCs infected with control (ctrl) or miR‐181a‐5p overexpression (181a OE) viruses. (g,h) Western blot analysis of PTEN (g), p‐AKT and AKT (h) expression in early‐passage NSCs infected with control (ctrl) or miR‐181a‐5p sponge (181a sp) viruses. (e,f) were tested in late‐passage NSCs; (g,h) were tested in early‐passage NSCs. *p < 0.05, **p < 0.01, ***p < 0.001, ns: not significant. Values are presented as mean ± SD. Two‐way ANOVA with Tukey's post hoc test for multiple comparisons was applied in (c), while Student's t test was used in (d–h). See also Figure S4.

To study how miR‐181a‐5p regulates NSC proliferation, we used the miRDB and miRWalk databases to predict the potential target genes of miR‐181a‐5p and then compared those target genes with aging‐related genes from Aging Atlas (AA) gene set (Aging Atlas, 2021). We found that PTEN, a negative regulator of AKT signaling, was the potential target of miR‐181a‐5p under this condition (Figure 4a). As there are three miR‐181a‐5p binding sites in the 3′UTR of PTEN (Figure 4b), to confirm the binding between miR‐181a‐5p and PTEN, we cloned the wild‐type (WT) and mutated 3′UTR of PTEN into a pGL3 vector and performed a luciferase assay. The results showed that miR‐181a‐5p suppressed luciferase activity in the WT but not the mutated reporter. Furthermore, we found that site 1 and site 2 were important for miR‐181a‐5p binding, as miR‐181a‐5p could not suppress luciferase activity when site 1 and site 2 were mutated (Figure 4c), and the miR‐181a‐5p sponge or inhibitors could abolish the inhibitory effect of miR‐181a‐5p on luciferase activity, further confirmed the binding between miR‐181a‐5p and PTEN (Figure S4a,b). As the protein level of PTEN in the late‐passage NSCs was higher than that in the early‐passage NSCs (Figure 4d), we overexpressed miR‐181a‐5p in late‐passage NSCs and found a corresponding reduction in the PTEN protein level and an increase in the phospho‐AKT (p‐AKT) level (Figure 4e,f). In contrast, inhibition of miR‐181a‐5p in early‐passage NSCs upregulated the protein level of PTEN and downregulated the level of p‐AKT (Figure 4g,h). These results confirmed that the PTEN‐AKT pathway can be regulated by miR‐181a‐5p. Furthermore, we established PTEN knockdown and overexpression NSCs, respectively, and the expression of PTEN was validated by Western blotting (Figure S4c). Immunofluorescence staining analysis showed that knockdown of PTEN in late‐passage NSCs increased the percentage of Ki67/Sox2 or BrdU/Sox2 double‐positive cells (Figure S4d,e), while overexpression of PTEN in early‐passage NSCs reduced the percentage of Ki67/Sox2 or BrdU/Sox2 double‐positive cells (Figure S4f,g). Taken together, the above data show that PTEN suppresses NSC proliferation, which is contrary to the function of miR‐181a‐5p; thus, we speculated that PTEN may act as a target of miR‐181a‐5p in NSC proliferation.

**FIGURE 5:**
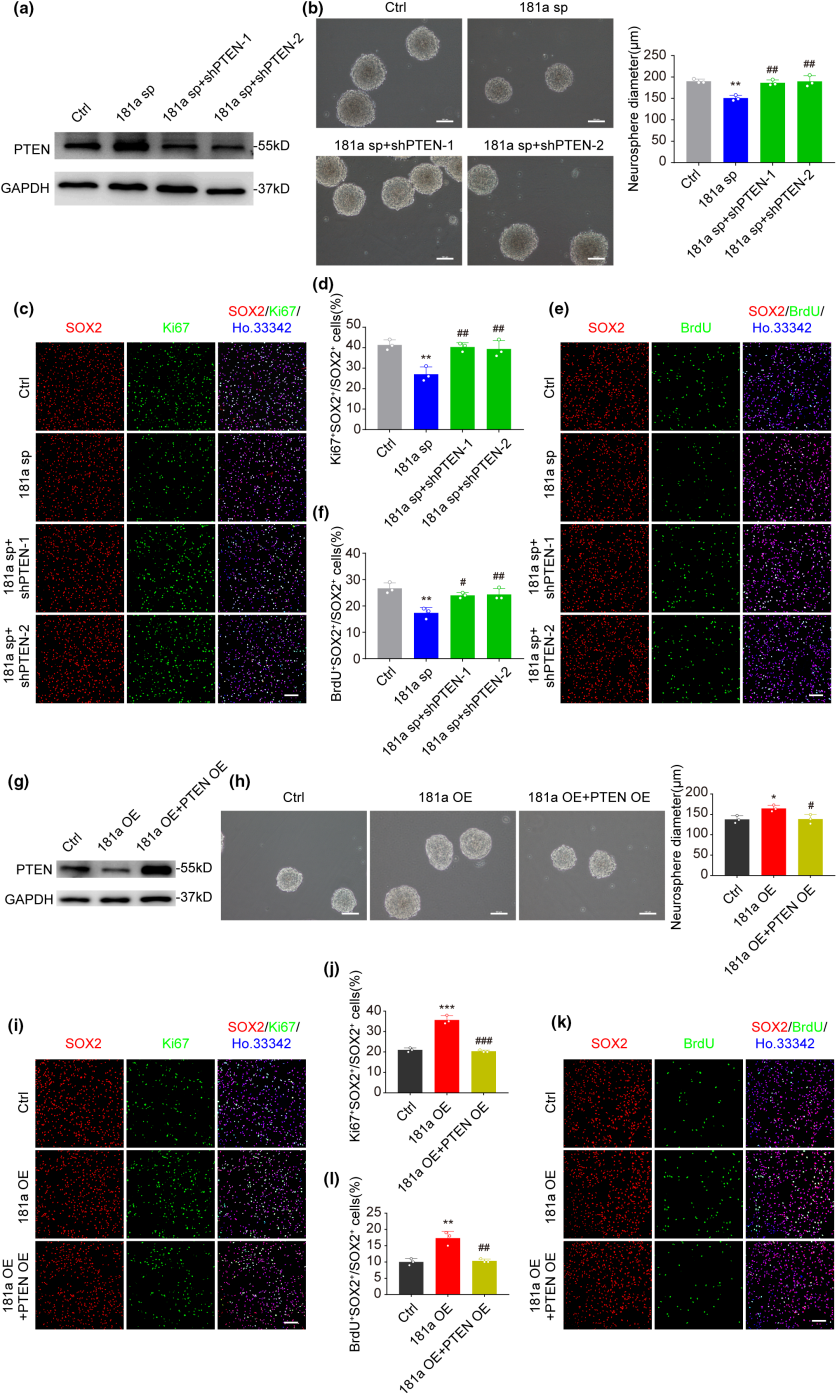
PTEN rescues NSC proliferation deficits induced by miR‐181a‐5p. (a) Western blot analysis of PTEN expression in early‐passage NSCs infected with control (ctrl), miR‐181a‐5p sponge (181a sp) only or miR‐181a‐5p sponge together with shPTENs (181a sp + shPTEN‐1; 181a sp + shPTEN‐2) viruses. (b) Representative images of early‐passage neurospheres infected with ctrl, 181a sp only or 181a sp together with shPTENs viruses (left), and quantification of neurosphere diameters (right). Scale bars, 100 μm. (c,d) Representative images of Ki67 (green) and SOX2 (red) after early‐passage NSCs were infected with ctrl, 181a sp only or 181a sp together with shPTENs viruses (c) and the proportion of Ki67^+^SOX2^+^ cells among all SOX2^+^ cells (d). Scale bars, 100 μm. (e and f) Representative images of BrdU (green) and SOX2 (red) after early‐passage NSCs were infected with ctrl, 181a sp only or 181a sp together with shPTENs viruses (e) and the proportion of BrdU^+^SOX2^+^ cells among all SOX2^+^ cells (f). Scale bars, 100 μm. (g) Western blot analysis of PTEN expression in late‐passage NSCs infected with control (ctrl), miR‐181a‐5p overexpression (181a OE) only or miR‐181a‐5p together with PTEN overexpression (181a OE + PTEN OE) viruses. (h) Representative images of late‐passage neurospheres infected with ctrl, 181a OE only or 181a OE together with PTEN OE viruses (left) and quantification of neurosphere diameters (right). Scale bars, 100 μm. (i,j) Representative images of Ki67 (green) and SOX2 (red) after late‐passage NSCs were infected with ctrl, 181a OE only or 181a OE together with PTEN OE viruses (i), and the proportion of Ki67^+^SOX2^+^ cells among all SOX2^+^ cells (j). Scale bars, 100 μm. (k,l) Representative images of BrdU (green) and SOX2 (red) after late‐passage NSCs were infected with ctrl, 181a OE only or 181a OE together with PTEN OE viruses (k), and the proportion of BrdU^+^SOX2^+^ cells among all SOX2^+^ cells (l). Scale bars, 100 μm. (a–f) were tested in early‐passage NSCs; (g–l) were tested in late‐passage NSCs. Nuclei were stained with Hoechst 33342 (blue), and “merge” images indicate the merging of images acquired with distinct channels (green, red and blue). *p < 0.05, **p < 0.01, ***p < 0.001 versus ctrl (b, d, f, h, j and l); ^#^ *p* < 0.05, ^##^ *p* < 0.01, ^###^ *p* < 0.001 versus 181a sp (b, d and f), or versus 181a OE (h, j and l). Values are presented as mean ± SD. One‐way ANOVA with Tukey's post hoc test for multiple comparisons was applied in (b), (d), (f), (h), (j), and (l).

To confirm this, PTEN was knocked down with shPTEN lentivirus in 181a sp NSCs (Figure 5a). The PTEN knockdown significantly rescued the inhibitory effect of 181a sp on the size of neurospheres, as well as the percentage of Ki67/Sox2 or BrdU/Sox2 double‐positive cells (Figure 5b–f). Next, we overexpressed PTEN in 181a‐overexpressing NSCs (Figure 5g) and found that the overexpression of PTEN in 181a OE NSCs also significantly reduced the influence of miR‐181a‐5p on the size of neurospheres as well as the percentage of Ki67/Sox2 or BrdU/Sox2 double‐positive cells (Figure 5h–l). In conclusion, these studies revealed that PTEN is the functional target of miR‐181a‐5p in NSC proliferation.

### 2.4 Inhibition of miR‐181a‐5p decreases the proliferation of NSCs and impairs the learning and memory abilities of young mice

We then explored whether miR‐181a‐5p is required for the proliferation of NSCs and the learning and memory abilities of young mice. rAAVs containing Nestin promoter‐driven miR‐181a‐5p sponge and GFP (Nes‐181a sp) were injected into the hippocampi of young mice (Figure S5a). GFP^+^ NSCs in the dentate gyrus of young mice were isolated by FACS, and the expression level of the miR‐181a‐5p sponge was measured by qRT–PCR (Figure S5b). The number of GFP^+^Ki67^+^, GFP^+^BrdU^+^ NSCs, and BrdU^+^DCX^+^ newborn neurons was decreased in the dentate gyrus of young mice in Nes‐181a sp group (Figure S5c–f), while the percentage of BrdU^+^DCX^+^ in total BrdU^+^ cells was not altered (Figure S5g). These data indicate that inhibition of miR‐181a‐5p decreases NSC proliferation but does not affect neuronal differentiation. In addition, in the NOR test, the discrimination index and the discrimination ratio of novel objects were lower in miR‐181a‐5p inhibition mice (Figure S5h–j), and in the MWM test, the miR‐181a‐5p inhibition mice located the hidden platform more slowly, crossed the platform fewer times, and spent less time in the goal quadrant than control mice (Figure S5k–p). These results suggest that miR‐181a‐5p is critical for the proliferation of NSCs and the learning and memory abilities of young mice.

### 2.5 MiR‐181a‐5p promotes the proliferation of NSCs and ameliorates learning and memory deficits in aged mice

**FIGURE 6:**
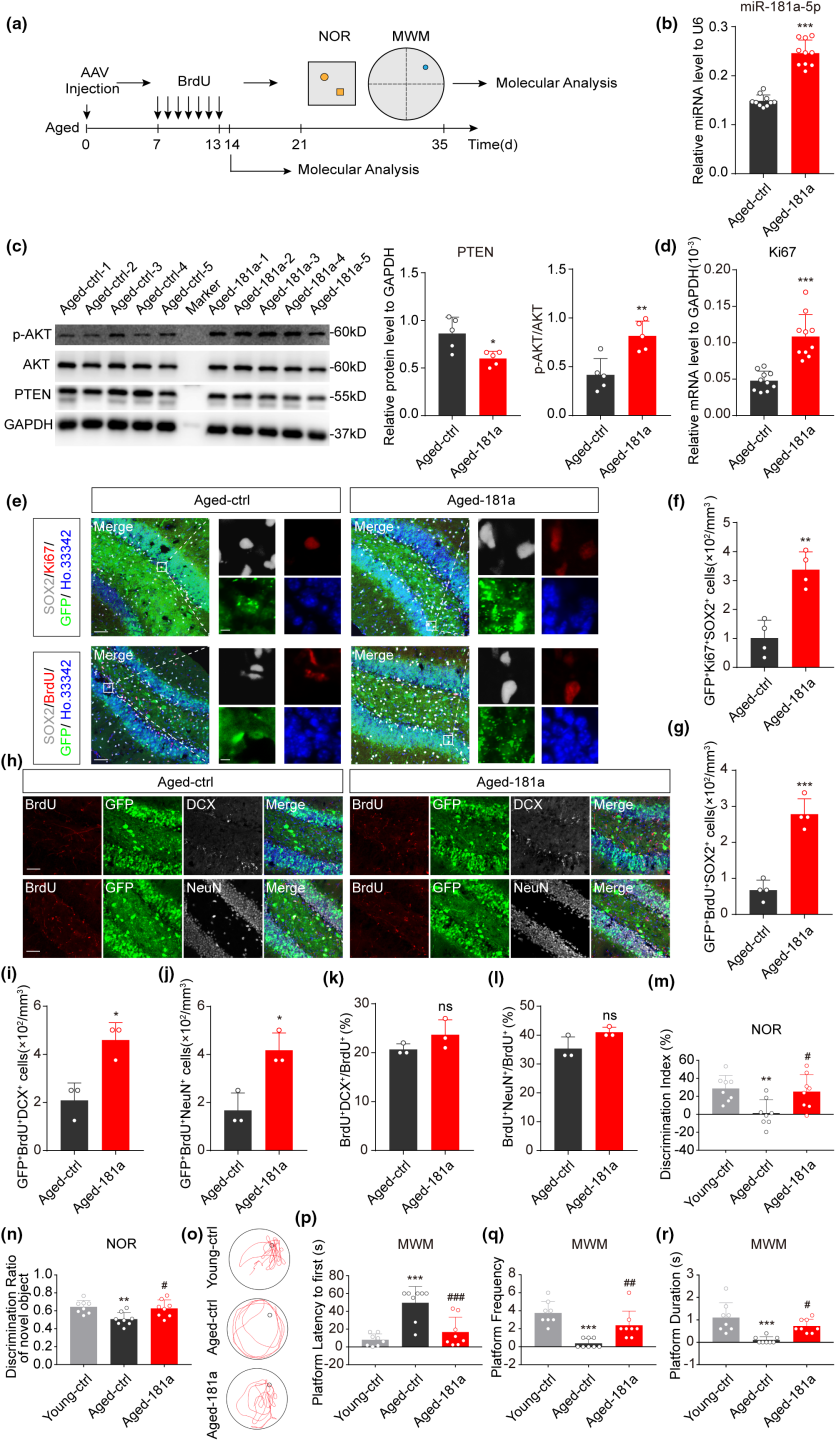
MiR‐181a‐5p promotes NSC proliferation in the hippocampus and ameliorates learning and memory deficits in aged mice. (a) Schematic diagram of the experimental design. (b) qRT–PCR analysis of the expression of miR‐181a‐5p in the hippocampi of aged‐ctrl and aged‐181a mice after intrahippocampal injection. U6 was used as the internal control (*n* = 10 per group). (c) Western blot analysis of PTEN, phospho‐AKT (p‐AKT), and AKT expression in the hippocampi of aged‐ctrl and aged‐181a mice. Shown are sample Western blot images and quantifications (*n* = 5 per group). (d) qRT–PCR analysis of the expression of Ki67 in the hippocampi of aged‐ctrl and aged‐181a mice. GAPDH was used as the internal control (*n* = 10 per group). (e–g) Representative images of GFP (green), Ki67 (red), BrdU (red), SOX2 (white), and Ho.33342 (blue) in the DG of aged‐ctrl and aged‐181a mice (e) and quantification of the number of GFP^+^Ki67^+^SOX2^+^Ho.33342^+^ cells (f) and GFP^+^BrdU^+^SOX2^+^Ho.33342^+^ cells (g) in the DG. Scale bars, 50 μm (left) and 5 μm (right) (*n* = 4 per group). (h) Representative images of GFP (green), BrdU (red), DCX (white), NeuN (white), and Ho.33342 (blue) in the DG of aged‐ctrl and aged‐181a mice. Scale bars, 50 μm. (i–l) Quantification of the number of BrdU^+^DCX^+^ (i) and BrdU^+^NeuN^+^ cells (j) in the DG and the proportion of BrdU^+^DCX^+^ (k) and BrdU^+^NeuN^+^ cells (l) among all BrdU^+^ cells (*n* = 3 per group). (m,n) Analysis of the NOR test. Discrimination index (m) and discrimination ratio of novel objects (n) (*n* = 8 per group). (o–r) Analysis of the MWM test. Representative traces were recorded with a video‐tracking system (o). Latencies to first reach the platform region (p), frequencies of crossing (q), and duration in the goal quadrant (r) were analyzed (*n* = 8 per group). *p < 0.05, **p < 0.01, ***p < 0.001 versus aged‐ctrl (b, c, d, f, g, and i–l), or versus young‐ctrl (m, n, and p–r); ^#^ *p* < 0.05, ^##^ *p* < 0.01 versus aged‐ctrl (m, n and p–r); ns: not significant. Values are presented as mean ± SD. Student's t test was used in (b–d), (f), (g), and (i–l), while one‐way ANOVA with Tukey's post hoc test for multiple comparisons was applied in (m), (n), and (p–r). AAV, adeno‐associated virus; DG, dentate gyrus; MWM, Morris water maze; NOR, novel object recognition. See also Figures S5, S6 and S7.

As we observed decreased expression of miR‐181a‐5p (Figure 1e), increased PTEN protein levels and decreased p‐AKT levels in the hippocampi of aged mice (Figure S6a), we further investigated whether miR‐181a‐5p regulated the proliferation of hippocampal NSCs in aged mice. For this purpose, adeno‐associated viruses (AAVs) overexpressing miR‐181a‐5p were injected into the hippocampi of 14‐month‐old mice (Figure 6a and S6b). The expression of miR‐181a‐5p increased in the hippocampus after 2 weeks of injection (Figure 6b). Western blotting showed decreased PTEN protein levels and increased p‐AKT levels in the miR‐181a‐5p overexpression group (Figure 6c). The mRNA level of Ki67 and the number of Ki67^+^Sox2^+^GFP^+^ or BrdU^+^Sox2^+^GFP^+^ NSCs were significantly higher in the dentate gyrus of aged mice overexpressing miR‐181a‐5p (Figure 6d–g), while the volume of the hippocampus was unchanged (Figure S3c), suggesting that miR‐181a‐5p can promote the proliferation of NSCs in aged mice by repressing PTEN signaling. Further, we found that the numbers of GFP^+^BrdU^+^DCX^+^ and GFP^+^BrdU^+^NeuN^+^ cells increased in the miR‐181a‐5p overexpression group (Figure 6h–j). However, the percentages of BrdU^+^DCX^+^ and BrdU^+^NeuN^+^ in total BrdU^+^ cells were not altered (Figure 6k,l), suggesting that overexpression of miR‐181a‐5p did not affect neuronal differentiation. Together, these results show that miR‐181a‐5p can increase the number of newborn neurons by increasing the number of NSCs but not directly promoting neuronal differentiation.

Furthermore, we performed behavior tests to assess the contribution of miR‐181a‐5p to the learning and memory abilities of aged mice. In the NOR test, the discrimination index and the discrimination ratio of novel objects were higher in miR‐181a‐5p‐overexpressing mice than those of aged‐control mice (Figure 6m,n and S6d), and in the MWM test, the miR‐181a‐5p overexpression mice located the hidden platform more quickly, crossed the platform more times, and spent more time in the goal quadrant than aged‐control mice (Figure 6o–r, Figure S6e,f). These results indicate that miR‐181a‐5p can ameliorate learning and memory deficits of aged mice.

In order to further confirm the specific regulatory role of miR‐181a‐5p in NSCs, the rAAVs containing Nestin promoter‐driven miR‐181a‐5p and GFP, which specifically infect NSCs, were injected into the dentate gyrus of aged mice (Figure S6g). GFP^+^ NSCs in the dentate gyrus of aged mice were isolated by FACS, and the overexpression efficiency of miR‐181a‐5p in GFP^+^ NSCs was validated by qRT–PCR (Figure S6h). Furthermore, the numbers of GFP^+^Ki67^+^ NSCs, GFP^+^BrdU^+^ NSCs, and BrdU^+^DCX^+^ newborn neurons was increased in the dentate gyrus of aged mice overexpressing miR‐181a‐5p (Figure S6i–l), but the percentage of BrdU^+^DCX^+^ in total BrdU^+^ cells was unchanged (Figure S6m), indicating that miR‐181a‐5p can increase the number of NSCs but does not affect neuronal differentiation. In addition, consistent with the learning and memory improvements observed earlier, the discrimination index and the discrimination ratio of novel objects were higher in Nestin‐miR‐181a‐5p‐overexpressing mice than in control mice in the NOR test (Figure S6n–p), and the Nestin‐miR‐181a‐5p overexpression mice located the hidden platform more quickly, crossed the platform more times, and spent more time in the goal quadrant than control mice in the MWM test (Figure S6q–v). As a recent study showed that AAV may ablate hippocampal neurogenesis (Johnston et al., 2021), a virus‐free miRNA agomir was injected into the hippocampi of aged mice to overexpress miR‐181a‐5p (Figure S7a). Similar phenotypes were observed with previous ones with regard to NSCs proliferation and learning and memory abilities (Figure S7b–p). Thus, miR‐181a‐5p can ameliorate learning and memory deficits in aged mice by promoting NSC proliferation. To further examine the effect of miR‐181a‐5p on neurons, we also injected rAAVs containing Synapsin promoter‐GFP (Syn‐GFP) to specifically infect neurons in the dentate gyrus of aged mice. Behavioural results showed no significant difference between the syn‐ctrl and syn‐181a group mice by the NOR test and MWM test, indicating that overexpression of miR‐181a‐5p in neurons did not affect the learning and memory ability of aged mice (Figure S7q–z).

## 3 DISCUSSION

Neurogenesis in the hippocampus persists throughout life but undergoes an age‐related decline (Kuhn et al., 1996). Neurogenesis is one of the major factors affecting aging‐related cognitive abilities, and promoting NSC neurogenesis has been considered as a potential strategy for the treatment of age‐related cognitive decline (Berdugo‐Vega et al., 2020; Drapeau & Abrous, 2008; Sasaki et al., 2021). However, prematurely promoting neurogenesis may cause NSCs to precociously differentiate into neurons and become depleted, which may diminish neurogenesis over time (Zhang et al., 2019; Zhou et al., 2018). A previous study found that deleting milk fat globule‐epidermal growth factor (EGF) 8 (Mfge8) in early postnatal NSCs could lead to premature NSC activation and reduce the adult NSC pool, which in turn led to a reduced number of proliferating NSCs and a decreased level of adult dentate neurogenesis (Zhou et al., 2018), indicating that maintenance of NSC proliferation without diminishing neurogenesis may be an effective means to expand NSC pool. Our present study showed that miR‐181a‐5p could promote the proliferation of NSCs and that overexpression of miR‐181a‐5p in the hippocampal NSCs could ameliorate learning and memory impairments in aged mice, suggesting that miR‐181a‐5p has potential applications for treating learning disabilities in elderly individuals.

NSCs have the capacity to self‐renew and are multipotent. In some cases, promoting NSC proliferation may decrease the differentiation potential and vice versa. For example, overexpression of miR‐219 promotes NSC differentiation into neurons but reduces NSC proliferation substantially (Murai et al., 2016). In contrast, overexpression of miR‐184 or miR‐195 enhanced NSC proliferation but repressed differentiation by targeting Numblike (Numbl) and MBD1, respectively (Liu et al., 2010, 2013). Previous studies showed that inactivation of Numbl in the cortex impaired neuronal differentiation (Li et al., 2003), and absence of MBD1 caused deficits in adult neurogenesis and hippocampal function (Zhao et al., 2003), which may be the reason why these miRNAs repress neuronal differentiation. In this study, we found that miR‐181a‐5p targets PTEN to promote the proliferation of NSCs but miR‐181a‐5p did not affect their differentiation into neurons and astrocytes, implying that miR‐181a‐5p serves as an important modulator of NSC proliferation in the hippocampus.

Pten is a well‐known tumor suppressor gene, and recent studies have shown that Pten also has important roles in brain development. Conditional deletion of PTEN in embryonic CNS stem/progenitor cells significantly increased cell proliferation, decreased cell death, and enlarged cell size without disturbing neuronal differentiation potential (Groszer et al., 2001). In adult neural stem cells, conditional deletion of PTEN enhanced self‐renewal, resulting in increased olfactory bulb mass and enhanced olfactory function (Gregorian et al., 2009). Direct deletion of PTEN may cause progressive enlargement and an enlarged, histoarchitecturally abnormal brain (Groszer et al., 2001). Coincidentally, a recent study found markedly increased expression of PTEN in a family with hereditary primary microcephaly; mild PTEN overexpression in brain organoids led to reduced neural precursor proliferation and the formation of significantly smaller brain organoids with microcephaly like phenotypes (Dhaliwal et al., 2021; Oliveira et al., 2019). These studies indicate that PTEN is a dosage‐sensitive gene in the regulation of brain development. In our study, we found that inhibiting PTEN increased the proliferation of NSCs and that overexpressing miR‐181a‐5p promoted the proliferation of NSCs in the aged hippocampus by reducing the protein level of PTEN but did not increase the volume of the hippocampus (Figure S6c), showing the potential value of miR‐181a‐5p and PTEN in the treatment of NSC proliferation defects.

Previous studies have shown that reduced miR‐181a‐5p expression is associated with a series of age‐related disorders, including immune dysfunction (Kim et al., 2019; Lu, Li, et al., 2021), sarcopenia (Soriano‐Arroquia et al., 2016), and hearing loss (Zhang et al., 2013). For example, miR‐181a‐5p was downregulated in aged NK cells, which inhibited NK cell development by reducing the production of IFN‐γ and the cytotoxicity of NK cells (Lu, Li, et al., 2021). MiR‐181a was also downregulated in sarcopenia, an age‐related loss of skeletal muscle mass and function, and negatively regulated myotube size (Soriano‐Arroquia et al., 2016). Moreover, miR‐181a was also decreased in the brain of Alzheimer's disease mice (Wu et al., 2019), implying that miR‐181a plays an important, yet undiscovered role in age‐related disorders. Here, we found that the expression level of miR‐181a‐5p was decreased in the hippocampi of aged mice and that overexpression of miR‐181a‐5p contributed to the proliferation of NSCs by targeting PTEN, both in vitro and in vivo. These findings first confirmed the crucial role of miR‐181a‐5p in NSC proliferation and differentiation, and further verified the importance of miR‐181a‐5p in the process of aging.

Collectively, our findings demonstrated the roles of miR‐181a‐5p in NSC proliferation. Considering the high drug potential of miRNAs, miR‐181a‐5p may provide the basis for the development of drugs to treat the aging‐related neurological disorders.

## 4 MATERIALS AND METHODS

### 4.1 Mice

C57BL/6J mice were purchased from the B&K Universal Group Limited and housed in standard, pathogen‐free conditions at the Laboratory Animal Research Center of Tongji University. All procedures involving animals were approved by the Institutional Animal Care and Use Committee of Tongji University in accordance with the Guide for the Care and Use of Laboratory Animals (NIH). Young mice (male, 12–14 weeks old) and aged mice (male, 14–16 months old) were used for experiments.

### 4.2 Behavioral tests

Novel object recognition (NOR) test and Morris water maze (MWM) test were used to evaluate the learning and memory abilities. See the Supplementary methods and materials for details.

### 4.3 5‐bromo‐2′‐deoxyuridine (BrdU) injections

Mice were given a daily single intraperitoneal injection of BrdU (50 mg/kg of body weight) for 7 or 10 consecutive days. For NSC proliferation studies, brains were perfused 24 h after the last BrdU injection. For differentiation studies, brains were perfused 3 weeks after the last BrdU injection.

### 4.4 Virus

For adeno‐associated virus serotype 2 preparation (AAV2), AAV control or AAV miR‐181a‐5p OE plasmids, pAAV‐RC and pHelper (ratio of vectors at 1:1:1) were cotransfected into 293A cells. All the cells were harvested and resuspended in 2.5 mL of serum‐free DMEM after transfection for 72 h. Then, the cells were treated with 4 freeze/thaw cycles in a liquid nitrogen bath and 37°C water bath. The viral supernatant was collected after centrifugation at 10,000*g* for 10 min. The virus was purified by a ViraBind™ AAV Purification Kit according to the manufacturer's protocol. Viral titers were 10^9^ v.g./ml. The recombinant adeno‐associated virus serotype 2/9 (rAAV2/9) contained Nestin promoter and Synapsin promoter were provided by OBiO Company (https://www.obiosh.com/). Viral titers were 10^10^ v.g./ml.

### 4.5 Stereotactic injections

C57BL/6J mice were anesthetized by intraperitoneal injection of AVER and fixed in a stereotaxic frame. Then, the coordinates relative to the bregma were located as X = ±1.75 mm, Y = ‐1.75 mm, and Z = ‐2.06 mm by using an ultra‐precise stereotactic injector. The AAV (2 μL) was injected into the DG by a Hamilton syringe at a rate of 0.2 μL/min. 10 min after injection, the syringe was slowly pulled out to reduce the loss of virus. After injection of the bilateral DG, the skin was closed by medical suture, and then, the animal was placed on a heating pad until it regained consciousness. Proliferation‐related studies were performed 2 weeks post stereotaxic injections, behavioral tests were performed 3 weeks post stereotaxic injections, and neurogenesis‐related studies were performed 5 weeks post stereotaxic injections.

### 4.6 In situ hybridization

Fixed Frozen brains of 12‐week‐old mice were cut into 10 μm thickness and examined using the miRNAscope technique according to the manufacturer's protocol (Advanced Cell Diagnostics, https://acdbio.com/).

### 4.7 Fluorescence‐activated cell sorting (FACS)

The hippocampi were isolated after Nestin‐GFP or Synapsin‐GFP rAAVs injected and dissociated with the Neural Tissue Dissociation Kit P (Miltenyi Biotec). GFP^+^ cells were sorted directly into RNAiso (Takara). Flow cytometry was performed on a BD FACS AriaII instrument. The data were analyzed using FlowJo software.

### 4.8 Cell culture and differentiation

Embryonic NSCs were isolated from the forebrain of E13.5 C57BL/6J mouse embryos as previously described (Ahlenius & Kokaia, 2010). Adult hippocampal NSCs were isolated from the male C57BL/6J mice at 8‐week‐old with the Neural Tissue Dissociation Kit P according to the manufacturer's protocol (Miltenyi Biotec). NSCs were cultured as neurospheres in growth medium composed of DMEM/F12 (Gibco), 2% B27 without vitamin A (Invitrogen), 1% GlutaMAX (Invitrogen), 1% NEAA (Invitrogen), 1% sodium pyruvate (Invitrogen), 20 ng/mL EGF (Sino Biological), and 20 ng/mL FGF‐2 (Sino Biological).

NSCs were passaged every 4 days. Early‐passage NSCs refer to passages 5–8, and late‐passage NSCs refer to passages 19–22. To assess neurosphere diameter, NSCs were seeded in ultralow‐adhesion 6 cm dishes at a density of 6 × 10^5^ cells per dish. The neurospheres were captured on the fourth day, and their diameters were quantified with ImageJ software. To assess cell proliferation, NSCs were dissociated into single cells with Accutase (Gibco) and seeded in coverslips pretreated with polyornithine (Gibco) and lamimin (Sigma); BrdU was added for the last 2 h prior to cell fixation. To assess differentiation, NSCs were dissociated into single cells and seeded on coverslips or 6‐well plates (Corning) pretreated with polyornithine and laminin. The next day, EGF and FGF‐2 were removed. After 48 h, follow‐up experiments were conducted to evaluate differentiation into neurons and astrocytes.

### 4.9 Vector

For the miR‐181a‐5p sponge vector, 9 copies of the miR‐181a‐5p sponge sequence and WPRE were cloned into the Fuw vector (Addgene). For the shPTEN vector, short hairpin RNAs (shRNAs) targeting PTEN were cloned into the pLKO.1 vector (Addgene). For the luciferase reporter vector, full‐length Pten 3'UTR or 3'UTR with 1–3 mutant miR‐181a‐5p binding sites were cloned into the pGL3 vector (Addgene). Mutant vectors changed the miR‐181a‐5p target site in the PTEN 3'UTR from 5′‐TGAATGT‐ 3′ to 5'‐ACATTCA‐3′. For the AAV miR‐181a‐5p OE vector, the miR‐181a‐5p sequence, WPRE, ubiquitin promoter sequence, and a GFP fluorescent reporter were cloned into the AAV vector (VPK‐410, Cell Biolabs). All vectors were verified by DNA sequencing. Detailed primer sequences are listed in Table S1.

### 4.10 Lentiviral packaging, concentration, and infection

The lentiviral plasmids with Pax2 and VSVG (ratio of plasmids at 4:3:2) were cotransfected into 293FT cells. The medium containing the virus was filtered with a 0.45 μm filter and purified with Lenti‐concentin (5×) (Excell Bio) according to the manufacturer's protocol to remove the effect of serum on NSCs.

### 4.11 NSC proliferation curve

The neurospheres were dissociated and aliquoted into 24‐well plates pretreated with polyornithine and lamimin at a density of 6 × 10^4^ cells per well. The NSCs were infected with different viruses and counted by a cytometer (CountStar) at 24 h, 48 h, 72 h.

### 4.12 Dual‐luciferase reporter assay

Control Renilla luciferase plasmids, pGL3‐luciferase reporter plasmids, control, or miR‐181a‐5p mimics were cotransfected into NIH3T3 cells. Luciferase activity was measured by the Dual‐luciferase reporter assay system (Promega) after transfection for 48 h.

### 4.13 Quantitative RT–PCR

Brain tissues and cultured cells were lysed by RNAiso (Takara), total RNA was extracted by chloroform and isopropanol, and the quality of RNA was measured by the 260/280 ratio. Five hundred nanograms of total RNA was used to synthesize cDNA with the PrimeScript™ RT reagent Kit (Takara). qRT–PCR was performed using SYBR Green qRT–PCR Master Mix (Bio–Rad) on an Mx3000 instrument (Agilent). Relative expression levels were calculated by the 2^−ΔΔCt^ method with GAPDH expression as an internal control. MiRNA expression levels were measured using the Bulge‐Loop™ miRNA qRT–PCR Primer Set (RiboBio) according to the manufacturer's instructions. Relative expression levels were calculated with U6 expression as an internal control. Detailed primer sequences are listed in Table S1.

### 4.14 Western blotting

For the cultured cells, the same number of cells was collected and lysed in sodium dodecyl sulfate (SDS, Amersham) buffer with 1× protease inhibitor (PI, Roche). For brain tissues, the tissues were lysed with RIPA for 30 min on ice. The protein concentrations were calculated using a bicinchoninic acid assay (BCA). Equal amounts of proteins were separated by SDS–PAGE and transferred onto PVDF membranes. The membranes were blocked with 3% BSA at room temperature for 1 h and then incubated with primary antibodies diluted in TBST overnight at 4°C and the corresponding secondary antibodies for 1 h at room temperature. Blots were visualized by enhanced chemiluminescence (ECL). GAPDH was used as an internal control. ImageJ was used for Western blot grayscale analysis. The antibodies are listed in Table S2.

### 4.15 Statistical analysis

The data are presented as the means ± SD from three independent biological replicates. Student's *t* tests were used for two‐group comparisons; one‐way ANOVA and two‐way ANOVA followed by Tukey's post hoc test were used for multiple comparisons. *p* < 0.05 was considered statistically significant. ^*, #^ *p* < 0.05; ^**, ##^ *p* < 0.01; ^***, ###^ *p* < 0.001. ns: not significant.

## AUTHOR CONTRIBUTIONS

Q.Y.S., J.J.X, and J.H.K conceptualized and designed the project. Q.Y.S. performed the experiments, collected data, analyzed data, and prepared figures. L.M., Y.X.W., A.L.T, and Z.H.Y assisted in animal experiments. J.Q., X.W., and J.G.L. assisted in molecular biology experiments. Q.Y.S., J.J.X, J.H.K, and Y.K.W wrote the manuscript. J.H.K involved in funding acquisition and project administration, provided the resources, and supervised the study.

## CONFLICT OF INTEREST STATEMENT

The authors declare that they have no conflict of interest.

## Supporting information


Appendix S1.

## Data Availability

This manuscript does not include large datasets.
