# Supplementary material for: MiR‐181a‐5p promotes neural stem cell proliferation and enhances the learning and memory of aged mice
Source: Aging Cell. 2023 Feb 16;22(4):e13794. doi: 10.1111/acel.13794 (PMC10086527; doi:10.1111/acel.13794)
Supplement: Supplementary file 1 — Appendix S1. [file ACEL-22-e13794-s001.docx]

Supplementary Information for

**MiR-181a-5p promotes neural stem cell proliferation and enhances the learning and memory of aged mice**

Qiaoyi Sun^1^, Li Ma^1^, Jing Qiao^1^, Xing Wang^1^, Jianguo Li^1^, Yuxi Wang^1^, Ailing Tan^1^, Zihui Ye^1^, Yukang Wu^1^, Jiajie Xi^1 *^, and Jiuhong Kang^1 *^

1. Supplementary methods and materials
2. Appendix figures (Figures S1-7)
3. Appendix tables (Tables S1-3)

**Supplementary methods and materials**

**Immunostaining**

For immunostaining of the cultured cells, the cells were washed with PBS and fixed with 4% paraformaldehyde (PFA) for 15 min at 4 °C, washed three times with PBS to remove residual PFA and then incubated with blocking buffer containing 0.1% Triton X-100, 10% donkey serum and PBS for 1 h at room temperature. Subsequently, the cells were incubated with primary antibodies that were diluted in blocking buffer, placed in a wet box at 4 °C overnight and labeled with the secondary antibodies and Hoechst 33342. The cells were captured at 20× on a confocal microscope (Nikon). For BrdU incorporation, BrdU-pretreated cells were fixed with 4% PFA and treated with 2 M HCL for 20 min. Then, immunostaining was performed as described above. To quantify the immunofluorescence staining of cells expressing different cell markers, at least 9 sections from three independent experiments were assessed.

For mouse tissue processing, the mice were perfused with 4% PFA. Then the brains were dissected, rinsed with PBS and fixed in 4% PFA overnight. Over the next few days, the brains were dehydrated by successive placement in 15% sucrose and 30% sucrose until the brain had sunk. Subsequently, the brain tissues were embedded in Tissue-Tek O.C.T. compound and sliced at a thickness of 35 μm by a freezing microtome (Leica CM3050 S). The slices were stored at -20 °C for future immunostaining analysis. The brain sections were washed three times with PBS, incubated with blocking buffer, incubated with primary antibodies and labeled with the corresponding secondary antibodies. The brain sections were captured at 20×, 40× or 63× magnification on a confocal microscope (Leica SP8). For BrdU incorporation, the sections were treated with 2 M HCL for 45 min. Then, the tissue immunostaining methods described above were performed. The antibodies are listed in Table S2.

**Novel object recognition (NOR) test**

Mice were transferred to the experimental room to habituate one day in advance. The test included two stages: 1) Training phase: The training phase lasted three days and started at the same time every morning. During the experiment, mice were habituated to a black chamber with two identical objects fixed at equal distances at the bottom diagonal of the chamber and allowed to explore for 20 min. Then, the mice were returned to their home cage, and the chamber was cleaned with ethanol to prevent residual odors from affecting the behavior of other mice. 2) Testing phase: Twenty-four hours after the last training period, one of the objects was replaced by a new object that was equal in height to the old object but different in shape. The mice were allowed to freely move around for 6 min, and their behavior was recorded by cameras; exploration was defined as the mouse touching the objects with their nose or a paw. The exploration time was measured with two stopwatches for each mouse by watching the recorded behavior. The discrimination index (DI) = (novel-object exploration time – familiar-object exploration time) / total exploration time*100; this value is expressed as a percentage. The discrimination ratio (DR) = novel-object exploration time /total exploration time.

**Morris water maze (MWM) test**

Mice were transferred to the experiments room to habituate to the environment one week in advance. The maze apparatus consisted of a circular pool (120 cm in diameter) filled with water at 22±2 ℃ and a fixed platform (10 cm in diameter), with external location cues. The test included two stages: 1) Training phase: The mice were trained to locate the platform over four consecutive days. The pool was divided into 4 quadrants, and the mice were placed in the pool facing the wall at a fixed starting point in each quadrant. For each quadrant, the mice were allowed to locate the platform for a maximum of 60 s (the platform was placed 1 cm below the water surface, which was invisible to the mice), and the time it took the mouse to reach the platform was recorded. If the mouse found the platform within 60 s, it was allowed to rest on the platform for 20 s. If the mouse failed to find the platform within 60 s, it was placed on the platform for 20 s. 2) Testing phase: Twenty-four hours after the last training period, the platform was removed. The mice were placed into the water at the testing points. The swim path of the mice over 60 s were recorded by a video tracking system and analyzed by EthoVision XT software. The mouse spatial memory ability was assessed by the latency to locate the hidden platform, the number of times they crossed the hidden platform and the duration in the goal quadrant over 60 s.

**Nissl staining**

For the Nissl staining, ten sections were selected at equal intervals from each brain. The brain sections were mounted on glass slides, dried at room temperature and stained with toluidine blue (Solarbio, G1436). Then, the brain sections were slightly washed with distilled water and differentiated with 95% ethanol. Subsequently, the brain sections were sealed with neutral balsam and captured at 4× and 1.25× on an inverted microscope. The areas of the hippocampus for each brain section were determined by ImageJ. The total volume of the hippocampus was calculated by adding up the single volumes.

**MicroRNA mimics or inhibitor transfection**

Control/miR-181a-5p mimics, control/miR-181a-5p inhibitors and control-cy5/miR-181a-5p-cy5 inhibitors were purchased from RiboBio and dissolved based on the manufacturer’s protocol. For cell transfection, 5 μl of the dissolved samples and 3 μl of Fugene transfection reagent were added to 100 μl of Opti-MEM. After 20 min of incubation, the mixture was added dropwise to the cells.

**Appendix figures**

**
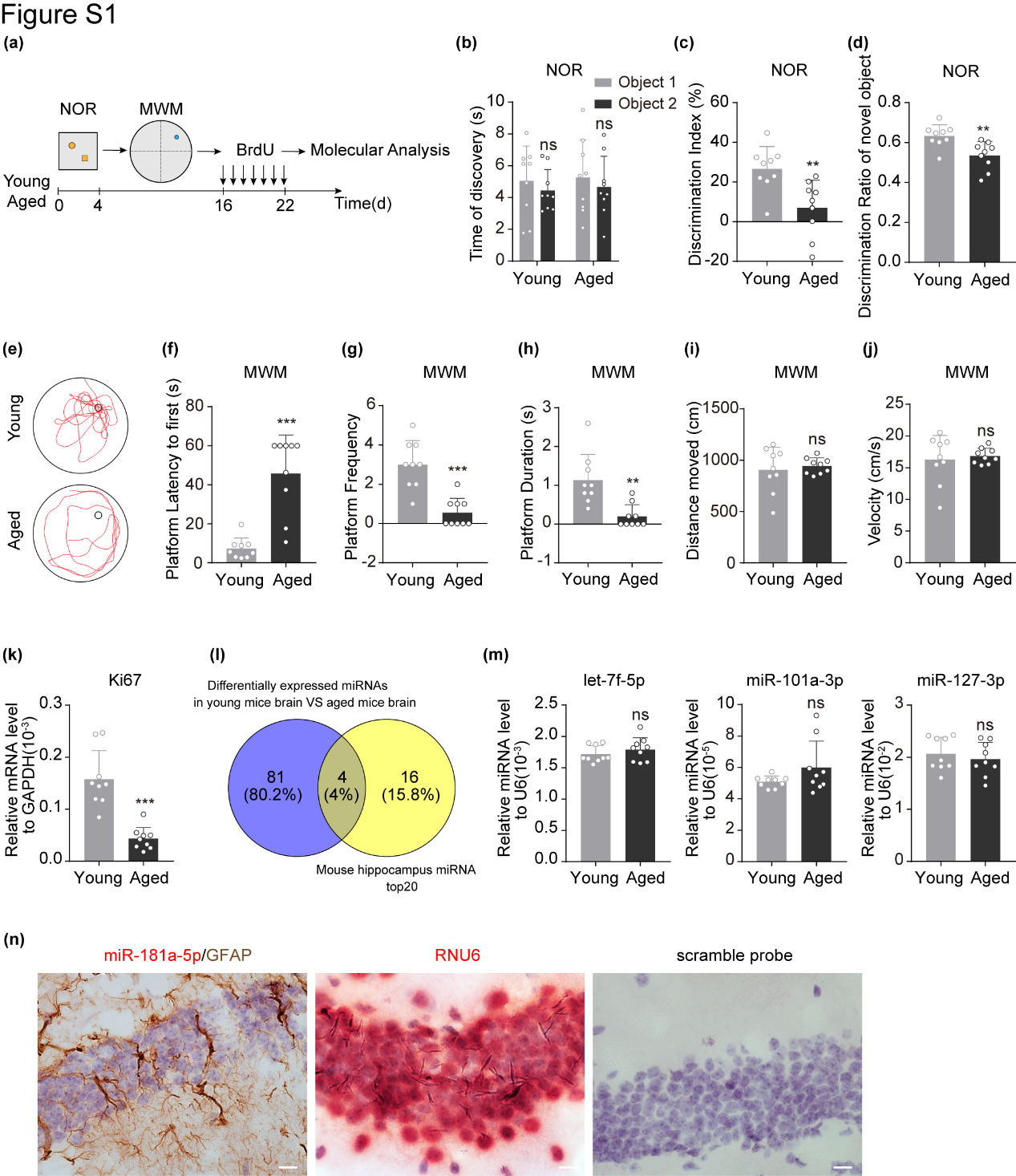
 Figure S1. Related to Figure 1.**

(a) Schematic diagram of the experimental design.

(b-d) Analysis of the NOR test. Exploration time for different objects in young and aged mice (b). Discrimination index (c) and discrimination ratio (d) of novel objects (n=9 per group).

(e-j) Analysis of the MWM test. Representative traces were recorded with a video-tracking system (e). Latencies to first reach the platform region (f), frequencies of crossing (g) and duration in the goal quadrant (h) were analyzed. Total swimming distance and speed of young and aged mice during the testing phase (i and j). (n=9 per group).

(k) qRT–PCR analysis of the expression of Ki67 in the hippocampus of young and aged mice. GAPDH was used as the internal control (n=9 per group).

(l) Venn diagrams showing the overlap of miRNA from GSE34393 and GSE107496.

(m) qRT–PCR analysis of the expression of let-7f-5p, miR-101a-3p and miR-127-3p in the hippocampus of young and aged mice. U6 was used as the internal control (n=9 per group).

(n) Representative images of miR-181a-5p in situ hybridization coupled to GFAP immunohistochemical staining in the dentate gyrus of 3-month-old WT mice. Representative images of positive and negative controls (RNU6 and scramble probes, respectively) are also shown. Scale bars: 5 μm.

^*^p < 0.05, ^**^p < 0.01, ^***^p < 0.001, ns: not significant. Values are presented as mean ± SD. Student’s t test was used in (c), (d), (f)-(k) and (m), while two-way ANOVA with Tukey’s post hoc test for multiple comparisons was applied in (b). MWM, Morris water maze; NOR, novel object recognition.


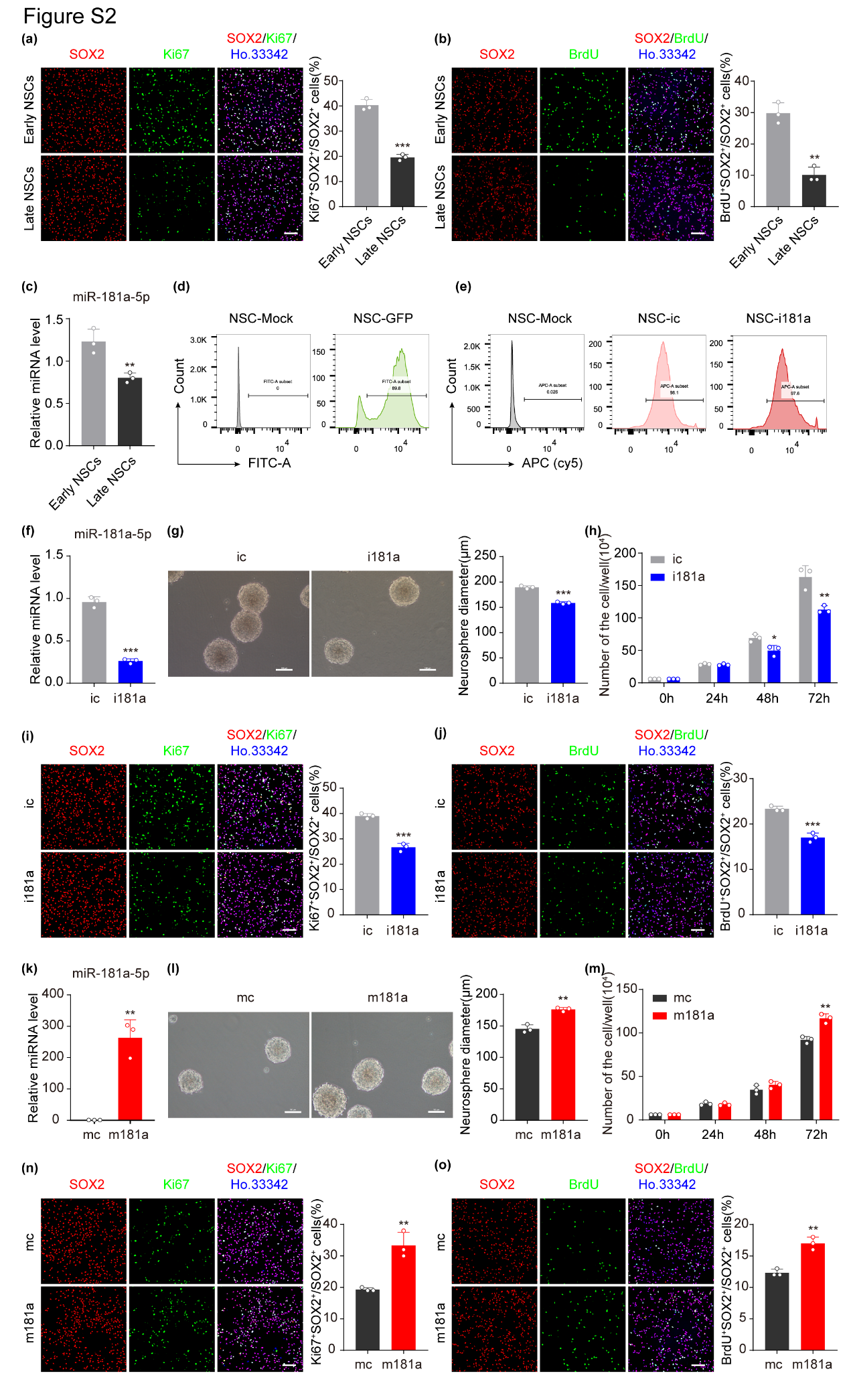


**Figure S2. Related to Figure 2.**

(a) Representative images of Ki67 (green) and SOX2 (red) in early-passage NSCs (early NSCs) and late-passage NSCs (late NSCs) (left) and the proportion of Ki67^+^SOX2^+^ cells among all SOX2^+^ cells (right). Scale bars, 100 μm.

(b) Representative images of BrdU (green) and SOX2 (red) in early NSCs and late NSCs (left) and the proportion of BrdU^+^SOX2^+^ cells among all SOX2^+^ cells (right). Scale bars, 100 μm.

(c) qRT–PCR analysis of the expression of miR-181a-5p in early NSCs and late NSCs. U6 was used as the internal control.

(d) Flow cytometry detection of percentage of GFP-positive cells.

(e) Flow cytometry detection of percentage of cy5-positive cells.

(f) qRT–PCR analysis the expression of miR-181a-5p in early-passage NSCs transfected with control inhibitor (ic) or miR-181a-5p inhibitor (i181a). U6 was used as the internal control.

(g) Representative images of early-passage neurospheres transfected with ic or i181a (left) and quantification of neurosphere diameters (right). Scale bars, 100 μm.

(h) Number of cells at 24 h, 48 h and 72 h after early-passage NSCs were transfected with ic or i181a.

(i) Representative images of Ki67 (green) and SOX2 (red) after early-passage NSCs were transfected with ic or i181a (left) and the proportion of Ki67^+^SOX2^+^ cells among all SOX2^+^ cells (right). Scale bars, 100 μm.

(j) Representative images of BrdU (green) and SOX2 (red) after early-passage NSCs were transfected with ic or i181a (left) and the proportion of BrdU^+^SOX2^+^ cells among all SOX2^+^ cells (right). Scale bars, 100 μm.

(k) qRT–PCR analysis of the overexpression efficiency of miR-181a-5p after late-passage NSCs were transfected with control mimics (mc) or miR-181a-5p mimics (m181a). U6 was used as the internal control.

(l) Representative images of late-passage neurospheres transfected with mc or m181a (left) and quantification of neurosphere diameters (right). Scale bars, 100 μm.

(m) Number of cells at 24 h, 48 h and 72 h after late-passage NSCs were transfected with mc or m181a.

(n) Representative images of Ki67 (green) and SOX2 (red) after late-passage NSCs were transfected with mc or m181a (left), and the proportion of Ki67^+^SOX2^+^ cells among all SOX2^+^ cells (right). Scale bars, 100 μm.

(o) Representative images of BrdU (green) and SOX2 (red) after late-passage NSCs were transfected with mc or m181a (left), and the proportion of BrdU^+^SOX2^+^ cells among all SOX2^+^ cells (right). Scale bars, 100 μm.

(f-j) were tested in early-passage NSCs; (k-o) were tested in late-passage NSCs. Nuclei were stained with Hoechst 33342 (blue), and “merge” images indicate the merging of images acquired with distinct channels (green, red and blue). ^*^p < 0.05, ^**^p < 0.01, ^***^p < 0.001. Values are presented as mean ± SD. Student’s t test was used in (a)-(c), (f), (g), (i)-(l), (n) and (o), while two-way ANOVA with Tukey’s post hoc test for multiple comparisons was applied in (h) and (m).


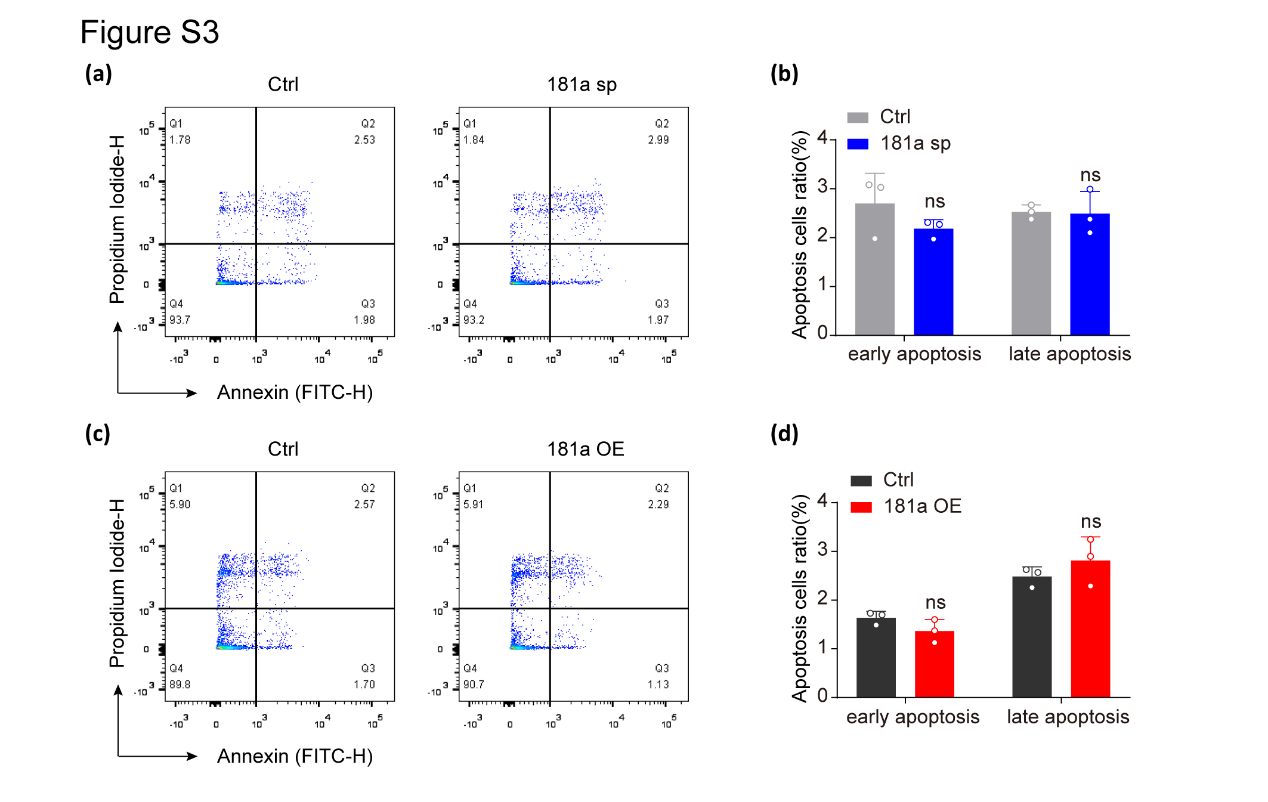


**Figure S3. Related to Figure 2.**

(a and b) Flow cytometry (a) and statistical results (b) of early-passage NSCs infected with control (ctrl) or miR-181a-5p sponge (181 sp) viruses.

(c and d) Flow cytometry (c) and statistical results (d) of late-passage NSCs infected with control (ctrl) or miR-181a-5p overexpression (181 OE) viruses.

ns: not significant. Values are presented as mean ± SD. Two-way ANOVA with Tukey’s post hoc test for multiple comparisons was used in (b) and (d).


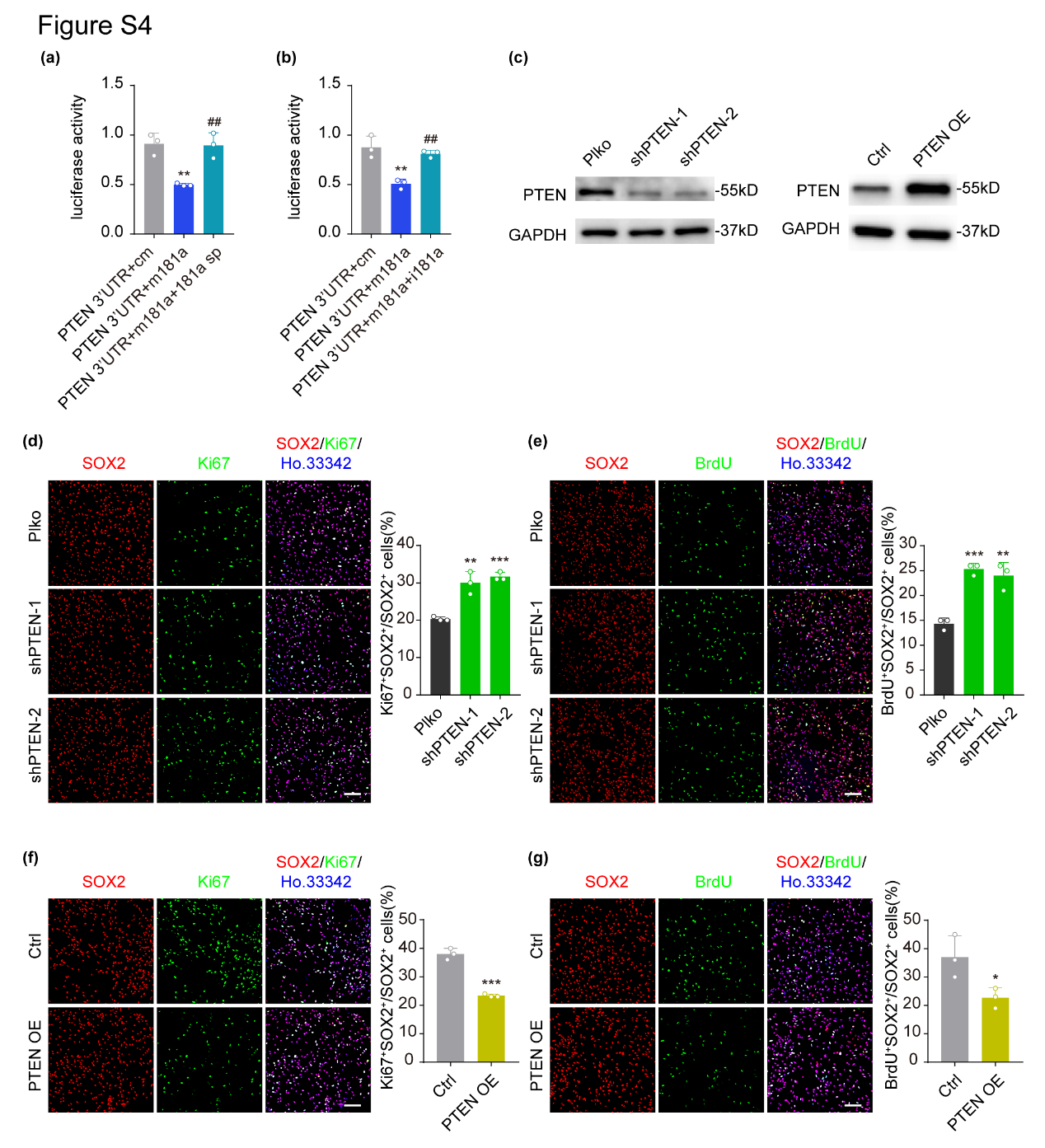


**Figure S4. Related to Figure 4.**

(a) Luciferase reporter assays for miR-181a-5p, Pten 3'UTR and miR-181a-5p sponge (181a sp).

(b) Luciferase reporter assays for miR-181a-5p, Pten 3'UTR and miR-181a-5p inhibitor (i181a).

(c) Western blot analysis of the knockdown (left) and overexpression (right) efficiency against PTEN.

(d) Representative images of Ki67 (green) and SOX2 (red) after late-passage NSCs were infected with plko or shPTENs viruses (left) and the proportion of Ki67^+^SOX2^+^ cells among all SOX2^+^ cells (right). Scale bars, 100 μm.

(e) Representative images of BrdU (green) and SOX2 (red) after late-passage NSCs were infected with plko or shPTENs viruses (left) and the proportion of BrdU^+^SOX2^+^ cells among all SOX2^+^ cells (right). Scale bars, 100 μm.

(f) Representative images of Ki67 (green) and SOX2 (red) after early-passage NSCs were infected with control (ctrl) or PTEN overexpression (PTEN OE) viruses (left) and the proportion of Ki67^+^SOX2^+^ cells among all SOX2^+^ cells (right). Scale bars, 100 μm.

(g) Representative images of BrdU (green) and SOX2 (red) after early-passage NSCs were infected with control (ctrl) or PTEN OE viruses (left), and the proportion of BrdU^+^SOX2^+^ cells among all SOX2^+^ cells (right). Scale bars, 100 μm.

(d and e) were tested in late-passage NSCs; (f and g) were tested in early-passage NSCs. Nuclei were stained with Hoechst 33342 (blue), and “merge” images indicate the merging of images acquired with distinct channels (green, red and blue). ^*^p < 0.05, ^**^p < 0.01, ^***^p < 0.001 versus Pten 3'UTR + cm (a and b), versus Plko (d and e) or versus Ctrl (f and g); ^##^P < 0.01 versus Pten 3'UTR + 181m (a and b). Values are presented as mean ± SD. One-way ANOVA with Tukey’s post hoc test for multiple comparisons was applied in (a), (b), (d) and (e), while Student’s t test was used in (f) and (g).


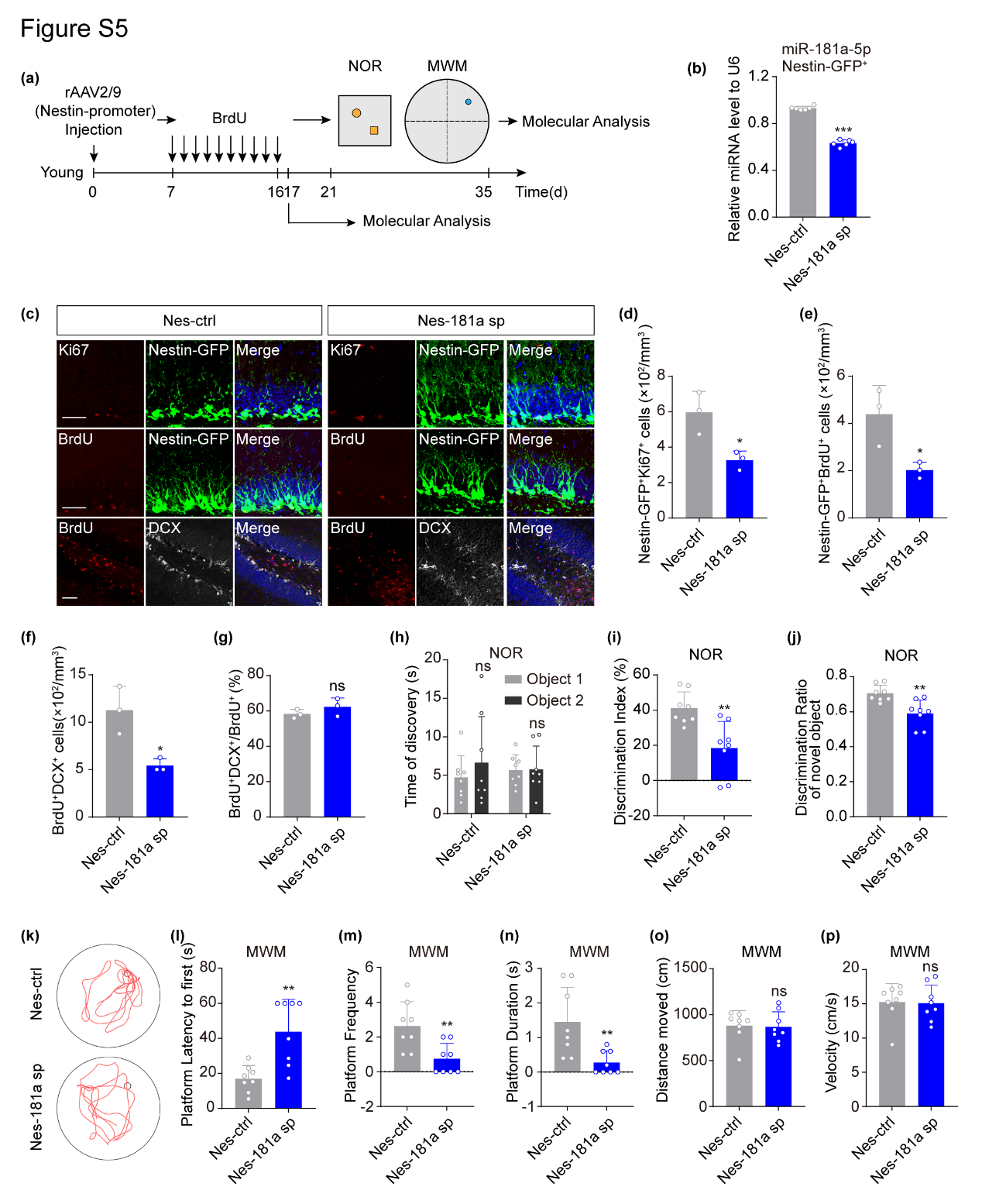


**Figure S5. Related to Figure 6.**

(a) Schematic diagram of the experimental design.

(b) qRT–PCR analysis of the expression of miR-181a-5p in Nestin-GFP^+^ populations sorted from the dentate gyrus of Nes-ctrl or Nes-181a sp mice. U6 was used as the internal control (n=6 per group).

(c) Representative images of Nestin-GFP (green), Ki67 (red), BrdU (red), DCX (white) and Ho.33342 (blue) in the DG of Nes-ctrl and Nes-181a sp mice. Scale bars, 50 μm.

(d-g) Quantification of the number of Nestin-GFP^+^Ki67^+^Ho.33342^+^ (d), Nestin-GFP^+^BrdU^+^ Ho.33342^+^ (e) and BrdU^+^DCX^+^ Ho.33342^+^ cells (f) in the DG and the proportion of BrdU^+^DCX^+^ cells among all BrdU^+^ cells (g) (n=3 per group).

(h-j) Analysis of the NOR test. Exploration time for different objects in Nes-ctrl and Nes-181a sp mice (h). Discrimination index (i) and discrimination ratio of novel objects (j) (n=8 per group).

(k-p) Analysis of the MWM test. Representative traces were recorded with a video-tracking system (k). Latencies to first reach the platform region (l), frequencies of crossing (m) and duration in the goal quadrant (n) were analyzed. Total swimming distance and speed of Nes-ctrl and Nes-181a sp mice during the testing phase (o and p) (n=8 per group).

^*^p < 0.05, ^**^p < 0.01, ^***^p < 0.001, ns: not significant. Values are presented as mean ± SD. Student’s t test was used in (b), (d)-(g), (i), (j) and (l)-(p), while two-way ANOVA with Tukey’s post hoc test for multiple comparisons was applied in (h). MWM, Morris water maze; NOR, novel object recognition.


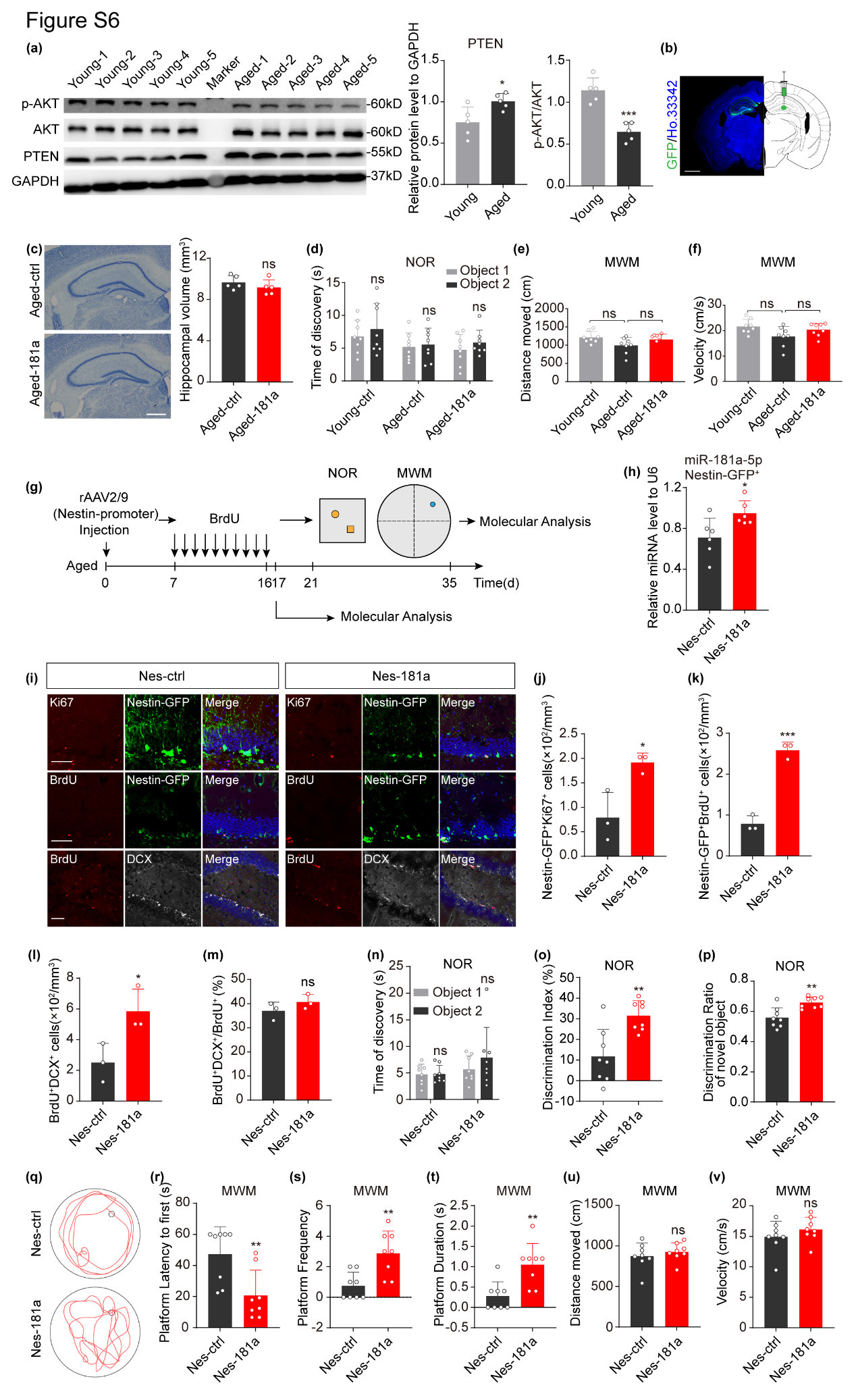


**Figure S6. Related to Figure 6.**

(a) Western blot analysis of PTEN, phospho-AKT (p-AKT) and AKT expression in hippocampus of young and aged mice. Sample Western blot images and quantifications are shown (n=5 per group).

(b) Experimental schematic of AAV intrahippocampal injection and representative images of GFP (green) and Ho.33342 (blue) in the brain. Scale bars, 1 mm.

(c) Nissl staining in the brain slices from aged-ctrl and aged-181a mice. Representative images are shown on the left; the volume of hippocampus are quantified on the right. Scale bars, 500 μm (n=5 per group).

(d) Analysis of the NOR test. Exploration time in different objects of young-ctrl, aged-ctrl and aged-181a mice (n=8 per group).

(e and f) Analysis of the MWM test. Total swimming distance and speed of young-ctrl, aged-ctrl and aged-181a mice during the testing phase (n=8 per group).

(g) Schematic diagram of the experimental design.

(h) qRT–PCR analysis of the expression of miR-181a-5p in Nestin-GFP^+^ populations sorted from the dentate gyrus of Nes-ctrl or Nes-181a mice. U6 was used as the internal control (n=6 per group).

(i) Representative images of Nestin-GFP (green), Ki67 (red), BrdU (red), DCX (white) and Ho.33342 (blue) in the DG of Nes-ctrl and Nes-181a mice. Scale bars, 50 μm.

(j-m) Quantification of the number of Nestin-GFP^+^Ki67^+^Ho.33342^+^ (j), Nestin-GFP^+^BrdU^+^Ho.33342^+^ (k) and BrdU^+^DCX^+^Ho.33342^+^ cells (l) in the DG and the proportion of BrdU^+^DCX^+^ cells among all BrdU^+^ cells (m) (n=3 per group).

(n-p) Analysis of the NOR test. Exploration time for different objects in Nes-ctrl and Nes-181a mice (n). Discrimination index (o) and discrimination ratio of novel objects (p) (n=8 per group).

(q-v) Analysis of the MWM test. Representative traces were recorded with a video-tracking system (q). Latencies to first reach the platform region (r), frequencies of crossing (s) and duration in the goal quadrant (t) were analyzed. Total swimming distance and speed of Nes-ctrl and Nes-181a mice during the testing phase (u and v) (n=8 per group).

^*^p < 0.05, ^**^p < 0.01, ^***^p < 0.001, ns: not significant. Values are presented as mean ± SD. Student’s t test was used in (a), (c), (h), (j)-(m), (o), (p) and (r)-(v), two-way ANOVA with Tukey’s post hoc test for multiple comparisons was applied in (d) and (n), while one-way ANOVA with Tukey’s post hoc test for multiple comparisons was applied in (e) and (f). MWM, Morris water maze; NOR, novel object recognition.


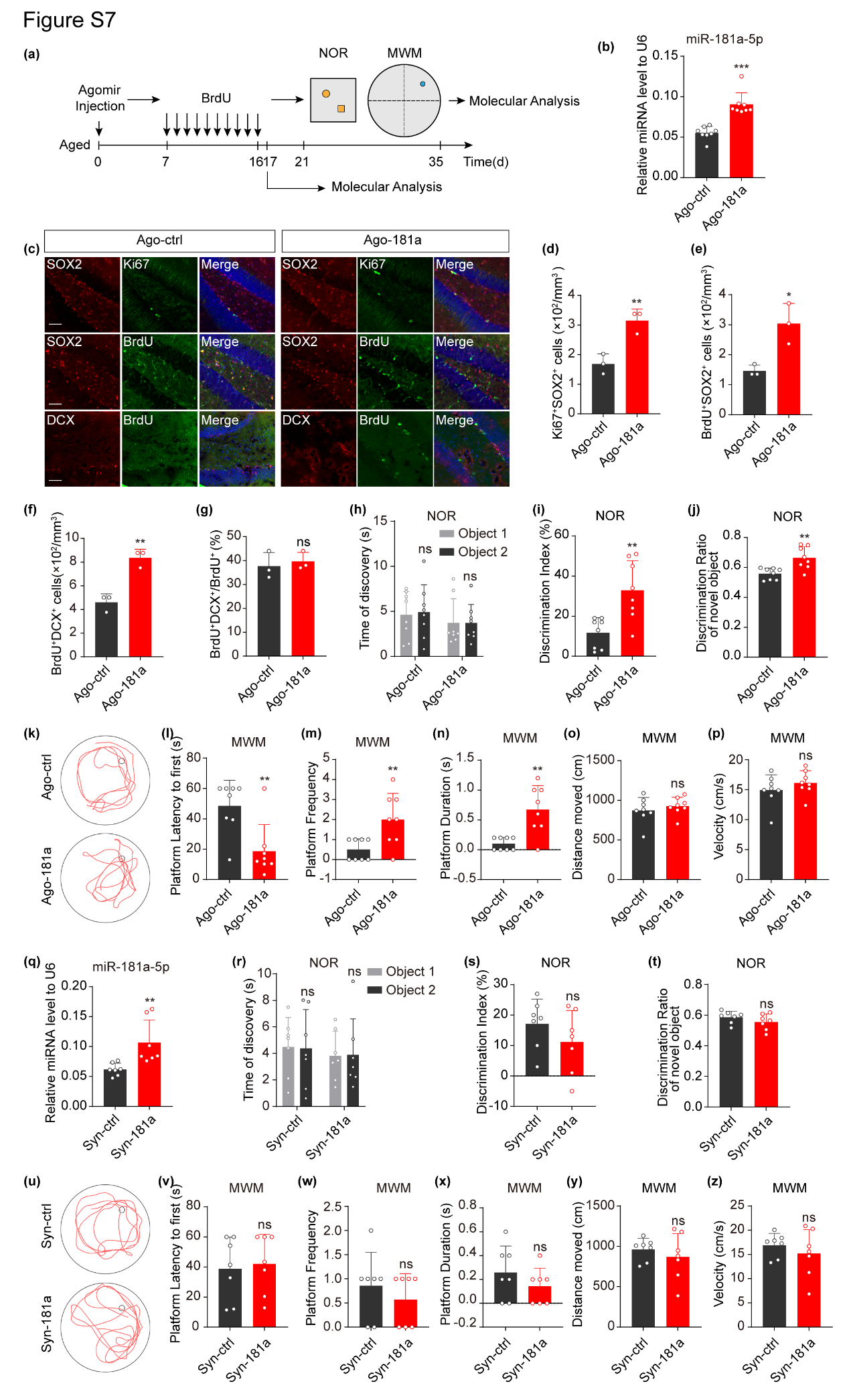


**Figure S7. Related to Figure 6.**

(a) Schematic diagram of the experimental design.

(b) qRT–PCR analysis of the expression of miR-181a-5p in the hippocampi of ago-ctrl and ago-181a mice after intrahippocampal injection. U6 was used as the internal control (n=8 per group).

(c) Representative images of Ki67 (green), BrdU (green), SOX2 (red), DCX (red), and Ho.33342 (blue) in the DG of ago-ctrl and ago-181a mice. Scale bars, 50 μm.

(d-g) Quantification of the number of SOX2^+^Ki67^+^Ho.33342^+^ (d), SOX2^+^BrdU^+^Ho.33342^+^ (e) and BrdU^+^DCX^+^Ho.33342^+^ cells (f) in the DG and the proportion of BrdU^+^DCX^+^ cells among all BrdU^+^ cells (g) (n=3 per group).

(h-j) Analysis of the NOR test. Exploration time for different objects in ago-ctrl and ago-181a mice (h). Discrimination index (i) and discrimination ratio of novel objects (j) (n=8 per group).

(k-p) Analysis of the MWM test. Representative traces were recorded with a video-tracking system (k). Latencies to first reach the platform region (l), frequencies of crossing (m) and duration in the goal quadrant (n) were analyzed. Total swimming distance and speed of Nes-ctrl and Nes-181a mice during the testing phase (o and p) (n=8 per group).

(q) qRT–PCR analysis of the expression of miR-181a-5p in the hippocampi of syn-ctrl and syn-181a mice after intrahippocampal injection. U6 was used as the internal control (n=7 per group).

(r-t) Analysis of the NOR test. Exploration time for different objects in ago-ctrl and ago-181a mice (r). Discrimination index (s) and discrimination ratio of novel objects (t) (n=7 per group).

(u-z) Analysis of the MWM test. Representative traces were recorded with a video-tracking system (u). Latencies to first reach the platform region (v), frequencies of crossing (w) and duration in the goal quadrant (x) were analyzed. Total swimming distance and speed of ago-ctrl and ago-181a mice during the testing phase (y and z) (n=7 per group).

^*^p < 0.05, ^**^p < 0.01, ^***^p < 0.001, ns: not significant. Values are presented as mean ± SD. Student’s t test was used in (b), (d)-(g), (i), (j), (l)-(q), and (s), (t) and (v)-(z). Two-way ANOVA with Tukey’s post hoc test for multiple comparisons was applied in (h) and (r). MWM, Morris water maze; NOR, novel object recognition.

**Appendix tables**

**Table S1. Primers used for this study.**

| Gene | Sequence (5’ to 3’) |
| --- | --- |
| **Primers used in vectors construction** | |
| miR-181a-5p sponge(3 repeats) | Forward:ACTCACCGACAGCATGAATGTTCCGACTCACCGACAGCATGAATGTTCCGACTCACCGACAGCATGAATGTT |
|  | Reverse:AACATTCATGCTGTCGGTGAGTCGGAACATTCATGCTGTCGGTGAGTCGGAACATTCATGCTGTCGGTGAGT |
| shPTEN-1 | Forward:CCGGAGACAAGGCCAACCGATACCTCGAGGTATCGGTTGGCCTTGTCTTTTTTG |
|  | Reverse:AATTCAAAAAAGACAAGGCCAACCGATACCTCGAGGTATCGGTTGGCCTTGTCT |
| shPTEN-2 | Forward:CCGGAGGTGAAGATATATTCCTCCAACTCGAGTTGGAGGAATATATCTTCACCTTTTTTG |
|  | Reverse:AATTCAAAAAAGGTGAAGATATATTCCTCCAACTCGAGTTGGAGGAATATATCTTCACCT |
| PTEN | Forward: CACAGACCGGTATGACAGCCATCATCAAAGAGATCGTTAG |
|  | Reverse: CCGGAATTCTCAGACTTTTGTAATTTGTGAATGCTGATCTTCA |
| **Primers used in qRT–PCR** | |
| Gapdh | Forward: GTGTTCCTACCCCCAATGTGT |
|  | Reverse: ATTGTCATACCAGGAAATGAGCTT |
| Ki67 | Forward: ATCATTGACCGCTCCTTTAGGT |
|  | Reverse: GCTCGCCTTGATGGTTCCT |
| WPRE | Forward: CCTTTTACGCTATGTGGATACGCTG |
|  | Reverse: CGTCAGCAAACA CAGTGCACACC |
| Map2 | Forward: GGTCACAGGGCACCTATTCA |
|  | Reverse: TGTTCACCTTTCAGGACTGC |
| Tubb3 | Forward: TAGACCCCAGCGGCAACTAT |
|  | Reverse: GTTCCAGGTTCCAAGTCCACC |
| Gfap | Forward: ACCAGCTTACGGCCAACAG |
|  | Reverse: CCAGCGATTCAACCTTTCTCT |
| S100β | Forward: TGGTTGCCCTCATTGATGTCT |
|  | Reverse: CCCATCCCCATCTTCGTCC |

**Table S2. Antibodies used for this study.**

| Antibodies | Source | Identifier |
| --- | --- | --- |
| anti-SOX2 | R&B system | AF2018 |
| anti-Ki67 | Abcam | Ab15580 |
| anti-BrdU | Abcam | Ab6326 |
| anti-BrdU | Bioworld | MB6004 |
| anti-MAP2 | Abcam | Ab32454 |
| anti-GFAP | Millipro | MAB360 |
| anti-GFP | Cell Signaling Technology | 2955S |
| anti-Dcx | Abcam | Ab18723 |
| anti-NeuN | Millipro | ABN78 |
| anti-Ho.33342 | Sigma | 14533 |
| anti-GAPDH | Bioworld | AP0063 |
| anti-PTEN | Santa | SC-7974 |
| anti-P-AKT | Cell Signaling Technology | 4060L |
| anti-AKT | Cell Signaling Technology | 2920S |
| Donkey  anti-Rabbit IgG 594 | Invitrogen | A21207 |
| Donkey  anti-Rabbit IgG 488 | Invitrogen | A21206 |
| Donkey  anti-Mouse IgG 594 | Invitrogen | A21203 |
| Donkey  anti-Mouse IgG 488 | Invitrogen | A21202 |
| Donkey  anti-Goat IgG 594 | Invitrogen | A11058 |
| Donkey  anti-Rat IgG 488 | Invitrogen | A21208 |
| Donkey  anti-goat IgG Cy5 | Jackson ImmunoResearch | 705-175-147 |
| Donkey  anti-Rabbit IgG Cy5 | Jackson ImmunoResearch | 711-175-152 |
| anti-Rabbit IgG HRP | Cell Signaling Technology | 7074S |
| anti-Mouse IgG HRP | Cell Signaling Technology | 7076S |
| Goat  anti-rabbit IgG HRP | Cell Signaling Technology | 8114P |
| Horse  anti-goat IgG HRP | Cell Signaling Technology | 63707S |

**Table S3. Potential target genes of miR-181a-5p in miRDB and miWalk.**

| **Potential target genes of miR-181a-5p from miRDB** |
| --- |
| Gpr22，Fmnl2，Ythdc2，Pi4k2b，Spry4，Fign，Gm14440，Ddx3x，1700066M21Rik，Nova1，Spire1，Chic1，Prtg，Prox1，Il1a，Phf20l1，Ppip5k2，Cpd，Klhl29，Ap1s3，Greb1l，Kmt2a，Larp4，Zfp781，Usp33，Dnajc13，Rnmt，Txndc12，Gskip，Wdr37，Zfp800，Palb2，E2f5，Pdap1，Tgfbr1，Osbpl3，Epc2，Bend3，Tcerg1，Sfmbt1，Slitrk1，Syne1，Man2a1，Gm14436，Ccp110，Ube2b，Gpsm1，Klhl5，D430041D05Rik，Ythdf3，Nr6a1，Tspan13，Lmo1，Btbd3，Cbx7，Klf15，Gse1，Cdc40，Lrrc32，Klf6，Crebrf，Ssx2ip，Rbbp7，Entpd6，Nus1，Wwc2，Cyp7a1，Prkcd，Cnot6l，Zfp280d，Cdyl，Cpne2，Dock4，Mb21d2，Zfp120，Acsl4，Thrb，Fam122b，Cops2，Pcdhac2，Gabra1，Taf9b，Mier3，Brd1，Cntn4，Caprin1，Tulp4，Rnf145，Clasp1，Rad21，Sfrs18，Elmsan1，Naa50，Lrba，Trak1，Esm1，Gfpt1，Zic3，Mtf2，Zfp97，Zfp960，Rnf34，Slc4a10，Tada2b，Snn，Anapc16，Fbxo33，Specc1l，Gpd1l，Cblb，Sec24a，Hipk3，Vcan，Afg3l2，Fktn，Ppp3r1，Fam3c，Usp42，Arnt2，Nfat5，Rbm46，Hoxa11，C77370，Mas1，Dnajc21，Bai3，Lox，Ralgapb，Zfp951，Mboat2，Sec24c，Kcna4，Srsf7，En1，Sgpp1，Ipo8，Tbc1d1，Carf，Hmbs，Cdc42bpa，Ap1g1，Ptbp3，Prom2，Med26，C2cd5，Zfp36l2，Lonrf2，Kank1，Fbxo3，Grik2，Ctdspl，Lyrm1，Twistnb，Derl1，Cdon，Gdap1，Pcdha7，Pcdha1，Per2，Pcdha11，Rlf，Mpp5，Pcdha3，Pcdha4，Ddx55，Rbm26，Rabgef1，Pcdhac1，Pcdha5，St8sia3，Lcorl，Lmbrd2，Pcdha8，Mfap3l，Ago2，Pcdha12，Pcdha6，Pcdha2，Tnfrsf11b，Pcdha9，Pkd1l1，Ss18l1，Cnksr3，Oxsm，Ankrd13c，Mtmr12，Acsl1，Carm1，Esr1，3110047P20Rik，Cpsf6，Mbtps2，Smcr8，Gatm，Birc6，Ywhag，Prrc2c，Galnt16，Hsp90b1，B3galt5，Mybl1，Jazf1，4921524J17Rik，Pdik1l，Grm5，Mtx3，Ercc5，Mfsd6，Dock7，Fam179b，Stxbp6，Clec10a，Dlg2，Wdr82，Adarb1，Eif4a2，Golga1，2310067B10Rik，Itsn2，Dynll2，Pde5a，Adamts1，Kcnq5，Mfsd1，Lclat1，Slc25a36，Rassf2，Ehd4，Tbpl1，Mlf1，Zbtb43，Adam11，Ubp1，Baz2b，Atp2b1，Nipal4，Hoxa1，Cnksr2，Pax9，Brwd1，Pdgfra，Klhl42，E2f7，Wasl，Tanc2，Api5，Wdr7，Ept1，Nr3c1，Kmt2c，Slc35f3，Ppp1r3c，Crim1，Cacnb2，Igf2bp2，Tsc22d2，Ccnk，Mkrn1，Ube3c，Notch2，8-Sep，Heca，Zfp869，Zfand6，Timp3，Trim2，Psap，Sowaha，Etnk1，Gm21949，Zdhhc7，Rras2，Mtpn，Ppp1r2，Ssr1，Plcl2，Synpr，Ino80，Tmem151b，9830147E19Rik，Abtb2，Zfp212，Cep97，Pbx1，Trnau1ap，Pam，Zfp317，Ankmy2，Spice1，Tnfaip1，Hic2，Proser1，Atxn3，Nptxr，Uri1，Glrb，Msi2，Fndc3a，Ankrd27，Rps6kb1，Ano1，Zbtb4，Sin3b，Nlk，Tgfbi，Sfr1，Ino80d，Sbno1，Fam83a，Atp11b，Slc18a2，Aftph，Akirin1，Prrg1，Cbx4，Metap1，Rufy3，Itga2，Pi15，Ncald，Ankrd44，Adam12，Mink1，Pnrc2，Tm9sf3，Sos1，Mex3b，Atg5，Ttpa，Dennd4c，Pgap1，Wnk1，Slc37a3，Bcl6b，Pawr，Mdh1b，Slc25a37，G3bp2，Fsd1l，Avl9，Lmo3，Naa15，Igsf11，Crebzf，Cyp2c39，Morc3，Fam178a，Rab11fip2，Pten，Ptbp2，Rsad1，Syn2，Dip2b，Tbc1d4，Dync1li2，Cep76，Etv6，Etohi1，Gpd2，Cxadr，Lrp12，Ccnj，Scd1，Aldh3a2，Papd5，Map2k1，Sun1，Arhgef3，Dpp8，Adm，Slc7a11，Acap2，Fam160a2，Gm5595，Pwp1，Ube2n，Fnip2，Dnaja4，Ccar1，Bhlhe40，Gm14306，Map1b，Ankrd49，Chmp1b，Dmxl2，Gm14431，Rab11a，Gm8898，Mllt10，Isca1，Yipf6，Dcun1d1，Cggbp1，Scyl3，Plekhj1，Cmpk2，Jmy，Rbm25，Phtf2，Gm14420，Zfp101，Creb1，Limch1，Gm2808，Sema4g，D3Bwg0562e，Akt3，Map1a，Trim30b，Unc5a，Zfp810，Acer3，Pdlim5，S1pr1，Tnpo1，Tox，Gm1564，Adamtsl1，Ppm1b，H2-K1，Tcf7l2，Unc80，Slc38a2，Elavl2，Agfg1，Fbxl3，Elavl4，Il7，Zfp600，Ubl3，Trpm3，Gm13242，Edem1，Gpbp1，Dlgap2，Cemip，2610044O15Rik8，Arl5a，Gls，Tmem185b，6720489N17Rik，Cyr61，Prnd |
| **Potential target genes of miR-181a-5p from miRWalk** |
| Whsc1l1，Myocd，Atxn1，Slc44a5，Rbm14，Slc6a5，St8sia2，Cd160，Prtg，Ptbp3，Vsig10l，G6pc2，Zmym2，Ctdspl，Umps，Atp6v1a，Tmem107，Ctcf，Mvd，Ephx3，Pdgfrb，Napg，Vsig4，L3mbtl3，Stx12，Camk2n1，Ubxn10，Rab11fip2，Erc1，Fn3k，Tmem18，Baz1a，Hspa9，Gucd1，Arhgef28，Hck，Kcnv2，Ccnc，Fuom，Ept1，Slc2a9，Notch2，Cmtm4，Cux1，Cldn15，Gabrg1，Synj1，Tsc1，Mrpl1，Etv3，Hn1，Whsc1，D5Ertd579e，Gprc5b，Ttc38，Rapgef6，Il1a，Fam53b，Nek3，Fnbp4，Trim44，Fbxo3，Aldh3b1，Pacs1，Atl3，Chrm1，Gm8369，Golga7，Fgfr1，Hook3，Galntl6，Palld，Psd3，Sugp2，E2f1，Pltp，Slc13a3，Rnf114，Sall4，Tshz2，Rcn1，Gpr176，Chp1，Ehd4，Lrrc57，Sema6d，Shc4，Galk2，Cbx3，Hoxa1，Jazf1，Tril，Tlx2，Tet3，Cyp26b1，Kcnc3，Gabra5，Klf13，Pde5a，Mettl14，Tram1l1，Papss1，Gbp7，Lpar3，Prkacb，Ptgfr，Lrriq3，Igf2bp1，Arhgap23，Ikzf3，Msl1，Ccr7，Psme3，Meox1，Mpp2，Aak1，Gfpt1，Antxr1，Mitf，Arl8b，Setd5，Slc6a11，Cxcl12，Fxyd4，Ret，Gja8，Man1a2，Lrig2，Capza1，Slc30a7，Sass6，Agl，Cnn3，Tmem266，Cd276，Celf6，Myo9a，Coro2b，Pias1，Megf11，Dennd4a，Pdcd7，Zfp609，Snx22，Tln2，Rora，Foxb1，Tcf12，Tex9，Mettl16，Crk，Tmem132e，Heatr6，Synrg，Usp32，Tbx2，Kif12，Tle1，Nfib，Sh3gl2，Dennd4c，Slc24a2，Mtap，Mier1，Acot11，Dio1，Zyg11b，Slco1a4，C2cd5，Fgfr1op2，Stk38l，Zfp444，Zim1，Rnf225，LOC108167466，Cers4，Sox1，Grk1，Champ1，Rbm41，Irs4，Amot，Magea5，Rps6ka3，Adgrg2，Olfr658，Cnga4，Lyve1，Usp47，Dkk3，1110004F10Rik，Plekha7，Rasgrp4，Cd22，Zfp715，Zfp658，Stam2，Acvr1c，Tanc1，Ifih1，Scn1a，Spc25，Ppig，Ubr3，Dlx1，Itga6，Rapgef4，Wipf1，Calcrl，Tfpi，Olfr1033，Rgr，Ghitm，Gm7995，Styx，Rnase6，Arhgef40，Dad1，Homez，Arhgap35，Ceacam5，Nlrp4e，Cadm4，Rhcg，Stard5，l7Rn6，Nars2，Rsf1，Spcs2，Arpp21，Acvr2b，Scn11a，Zfp651，Abhd5，Lars2，Lztfl1，Ccr2，H60c，Utrn，Tnfaip3，Map3k13，Eif4a2，Lpp，Trp63，Lrrc15，Senp5，Ubxn7，Lmln，Heg1，Poglut1，Pld6，Specc1，Atp1b2，Derl2，Dync1li2，D230025D16Rik，Nutf2，Pla2g15，Nfat5，Nudt7，Sdr42e1，Sstr3，Apobec3，Syngr1，Mief1，Adsl，Scube1，Pnpla3，1810041L15Rik，Ppara，Mlc1，Glo1，Slc37a1，Zfp799，Hspa1a，Tnf，Olfr111，Esp34，Mcc，Cep120，Aldh7a1，Slc12a2，Chsy3，Arhgef37，Onecut2，Spire1，Dcc，LOC108168393，Rnf165，8030462N17Rik，Slc14a1，Nfatc1，Zfp516，Timm21，Fam78b，Tmco1，Mgst3，Pbx1，Hsd17b7，Ddr2，Slamf7，Pigm，Srp9，Dusp10，Slc30a10，Angel2，Mfsd7b，Ppp2r5a，Ints7，Rcor3，Myl6，Pik3ip1，Limk2，Ccdc157，Ewsr1，Hus1，Sertad2，Bcl11a，Mafk，Iqce，Smurf1，Usp12，Mtif3，Nxph1，Ophn1，Snx12，Chic1，Zcchc18，Fam199x，Rnft2，Gm15800，Fam109a，Tctn1，Pptc7，Hcar1，Zfp664，Stx2，Vkorc1l1，Alkbh4，Rcan2，Hsp90ab1，Slc29a1，Klhdc3，Bysl，Uhrf1，Nfasc，Btg2，Ipo9，Pkp1，Lhx9，Rgs2，Ivns1abp，Lamc1，Xpr1，Pigc，Fmo1，Gorab，Slc19a2，Aldh5a1，Sox4，Irf4，Prpf4b，Cdyl，Ranbp9，Mcur1，Kif13a，Tpmt，Drd1，Pdlim7，Enpp1，Epb41l2，Ptprk，Tspyl1，Ddo，Sar1a，Tspan15，Egr2，Ado，1700040L02Rik，Pwwp2a，Adam19，Itk，Tgtp1，Tgtp2，Zfp354c，Rad50，Slc22a5，Csf2，Sparc，Gria1，Cnot8，Iba57，Guk1，Snap47，Hjurp，Arl4c，Hdac4，Kif1a，Tmem177，Ccdc93，Lypd1，Ubxn4，Elk4，Mfsd4a，Mmp25，Ntn3，Spsb3，Ube2i，Jmjd8，Rab11fip3，Kpna6，Khdrbs1，Arid1a，Clic4，Hnrnpr，Zbtb40，Slc25a34，Slit2，Nwd2，Tmem156，Apbb2，Tmem33，Bend4，Guf1，Corin，Fip1l1，Hopx，Adgrl3，Jchain，Efcab14，Uqcrh，Hectd3，Mpl，AU022252，Exo5，Ppt1，Mycl，Snip1，5730409E04Rik，Zfp933，Ubiad1，Exosc10，Gpr157，Errfi1，Tas1r1，Hes2，Trp73，Tprgl，Sdf4，Steap2，4921511H03Rik，Sri，Tmem243，Timp3，Parpbp，Tmpo，Dusp6，Trhde，Kcnmb4，Cpsf6，Ppm1h，Slc16a7，Shmt2，Itga2，Ube2e2，Zswim8，Chdh，Dcp1a，Sh3bp5，Mapk8，Gdf2，Nt5c2，Tdrd1，Ablim1，Gfra1，Cacul1，Pqbp1，Eras，Lancl3，Sytl5，Usp9x，Fundc1，Ibtk，Zic1，Slc25a36，Faim，Tmem108，Camkv，Ip6k2，Plxnb1，Atg2b，Mok，Zmynd11，Rala，Elf4，Zfp280c，Slitrk4，Cetn2，Atp2b3，Crybg3，Chmp2b，Cxadr，Adamts1，Adamts5，Map3k7cl，Krtap24-1，Tiam1，Tmem50b，Kcnj6，Map3k4，Acat3，Disc1，Itgb1，Msantd4，Yap1，Ccdc82，Zfp846，Pde4a，Ilf3，1810026J23Rik，Bmper，Opcml，Nfrkb，Ets1，Brd3，Gtf3c4，Set，Slc25a25，Zbtb43，Cntrl，Zbtb26，Rabgap1，Nr6a1，Smim7，Ednra，Inpp4b，Chd9，Ciapin1，Kdm5a，C3ar1，Cd4，Zfp384，Kcna6，Tigar，Parp11，Cracr2a，Clec2e，Clec9a，Olr1，Dusp16，Apold1，Gucy2c，BC049715，Arhgdib，Sgsh，Rptor，Nploc4，B3gntl1，Pdia6，Odc1，Id2，Cbll1，Pik3cg，Snx13，Ankmy2，Camkmt，Crem，Epc1，Aqp4，Dsc2，Asxl3，AW554918，Sft2d3，Gfra3，Fam53c，1700066B19Rik，Pcdha1，Pcdha3，Pcdha5，Pcdha6，Pcdha7，Pcdha8，Pcdha9，Pcdha10，Pcdha11，Pcdha12，Pcdhac1，Pcdhac2，Rdh10，Crispld1，Kcnq5，Fam135a，Phf3，Sema4c，Rnf149，Il18r1，Pou3f3，Tgfbrap1，Gulp1，Stk17b，Ankrd33b，Sema5a，Oxr1，Csmd3，Eif3h，Derl1，Zhx1，Nsmce2，Lrrc6，Chchd10，Wdr18，Midn，Sgta，Zbtb7a，D10Wsu102e，Polr3b，Ric8b，Hnrnpk，Golm1，Spata31d1b，Ctsj，Fancc，Zfp367，Rslcan18，Arrdc3，Atg10，Rasgrf2，Jmy，Wdr41，Fam169a，Mier3，LOC102633156，LOC102639653，Dido1，Dnajc5，Zbtb10，Zfp704，Pag1，Impa1，Tbl1xr1，Tnik，Eif5a2，Phc3，Gnb4，Usp13，Fam47e，11-Sep，Gm35911，Tmem150c，Arhgap24，Cdc7，Oasl2，Suds3，Fdft1，Ints9，Scara3，Bnip3l，Adamdec1，Hr，Cog3，Gm1587，Pcdh8，Rnf219，Gpc6，Dnajc3，Rap2a，Card6，Prkaa1，Zfr，6030458C11Rik，Sirpa，Prn，Prnd，Rassf2，Slc23a2，Plcb1，Pak7，Flrt3，Dzank1，Vsx1，4921509C19Rik，Srxn1，Nol4l，Klf9，Smc5，Vldlr，Pten，Tnks2，Arhgap19，Entpd7，Dnmbp，Btrc，Cflar，Nbeal1，Plekhm3，Lancl1，Igfbp5，Cnot9，Sgpp2，Ndufc1，Ufm1，Dclk1，Tm4sf1，Siah2，Med12l，Mbnl1，Mme，Plch1，Schip1，Kpna4，Sptssb，Bche，Map3k7，Srsf12，Rngtt，Zfp292，Aptx，Dnajb5，Arhgef39，Rgp1，Rnf38，Dcaf10，Tgfbr1，Rad23b，Tbcel，Scn2b，Apoa5，Gm4791，Usp28，Ttc12，Dlat，Sik2，Rdx，4930550C14Rik，Atm，Tlk2，Pitpnc1，Prkca，Kcnj2，Cd300lb，Tmem104，Ush1g，Gga3，Exoc7，Rbfox3，Prkcb，Arhgap17，Itgal，Zfp768，Zfp629，Tacc2，Ikzf5，Hmx2，Chst15，Slc11a2，Tfcp2，Pou6f1，Slc4a8，Eif4b，Sp1，Atf7，Cbx5，Cdip1，Ubn1，Rbfox1，Usp7，Zc3h7a，Hic2，Pi4ka，Armc10，Cnpy1，Otof，Ppm1g，Fosl2，Prr14l，Ywhah，Fgfr3，Crmp1，Foxa1，Actr10，Akap5，Slc39a9，Synj2bp-cox16，Zfp410，Eif2b2，Irf2bpl，Adck1，Dio2，Foxn3，Atxn3，Nrf1，Zc3hc1，D630045J12Rik，Braf，Wee2，Tcaf1，2810403A07Rik，Snx27，Anp32e，Otud7b，Kcnh1，Fam171a1，Prpf18，Celf2，Arl5b，Plxdc2，Arhgap21，Psd4，Zmynd19 |
